# Prognostic and predictive biomarkers for anti-EGFR monoclonal antibody therapy in *RAS* wild-type metastatic colorectal cancer: a systematic review and meta-analysis

**DOI:** 10.1186/s12885-023-11600-z

**Published:** 2023-11-16

**Authors:** Xiaona Lu, Yuyao Li, Yue Li, Xuemei Zhang, Jia Shi, Hai Feng, Zhuo Yu, Yueqiu Gao

**Affiliations:** 1 Department of Liver Disease, Shuguang Hospital Affiliated to Shanghai University of Traditional Chinese Medicine, Shanghai, 201203 China; 2 Institute of Infectious Disease, Shuguang Hospital Affiliated to Shanghai University of Traditional Chinese Medicine, Shanghai, 201203 China

**Keywords:** Colorectal cancer, Prognostic, Predictive value, Biomarkers, Anti-EGFR monoclonal antibody

## Abstract

**Background:**

*RAS* mutations affect prognosis in patients with metastatic colorectal cancer (mCRC) and have been identified as strong negative predictive markers for anti-epidermal growth factor receptor monoclonal antibody (anti-EGFR mAb) therapy, but many tumors containing wild-type RAS genes still do not respond to these therapies. Some additional biomarkers may have prognostic or predictive roles, but conclusions remain controversial.

**Methods:**

We performed a meta-analysis and systematic review of randomized controlled trials comparing anti-EGFR mAb therapy with alternative therapy that investigated the prognostic and predictive impact of additional biomarkers in *RAS* wild-type (wt) mCRC patients. Hazard ratios (HRs) and 95% confidence intervals (CIs) for progression-free survival (PFS) and overall survival (OS) and odds ratios (ORs) for objective response rate (ORR) were calculated. The prognostic value of biomarkers was investigated by separately pooling HR and OR for different treatment groups in an individual study. The predictive value was assessed by pooling study interactions between treatment effects and biomarker subgroups.

**Results:**

Thirty publications reporting on eighteen trials were selected, including a total of 13,507 patients. In prognostic analysis, *BRAF* mutations were associated with poorer PFS [HRs = 3.76 (2.47–5.73) and 2.69 (1.82–3.98)] and OS [HRs = 2.66 (1.95–3.65) and 2.45 (1.55–3.88)] in both the experimental and control arms; low *miR-31-3p* expression appeared to have longer PFS and OS. In terms of predictive effect, a lack of response to anti-EGFR therapy was observed in patients with *BRAF* mutant tumors (*P*_interaction_ < 0.01 for PFS). Patients with tumors with any mutation in the *KRAS*/*NRAS*/*BRAF*/*PIK3CA* gene also showed similar results compared with all wild-type tumors (*P*_interaction_ for PFS, OS, and ORR were < 0.01, < 0.01 and 0.01, respectively). While low *miR-31-3p* expression could predict PFS (*P*_interaction_ = 0.01) and OS (*P*_interaction_ = 0.04) benefit. The prognostic and predictive value regarding *PIK3CA* mutations, *PTEN* mutations or deletions, EGFR, EREG/AREG, *HER2*, *HER3*, and *HER4* expression remains uncertain.

**Conclusions:**

In *RAS* wt mCRC patients receiving EGFR-targeted therapy, *BRAF* mutation is a powerful prognostic and therapy-predictive biomarker, with no effect found for *PIK3CA* mutation, *PTEN* mutation or deletion, but the combined biomarker *KRAS*/*NRAS*/*BRAF*/*PIK3CA* mutations predict resistance to anti-EGFR therapy. Low *miR-31-3p* expression may have positive prognostic and therapy predictive effects. Evidence on the prognostic and predictive roles of EGFR and its ligands, and HER2/3/4 is insufficient.

**Supplementary Information:**

The online version contains supplementary material available at 10.1186/s12885-023-11600-z.

## Introduction

The epidermal growth factor receptor (EGFR) is a 170-kD transmembrane glycoprotein composed of three domains: an extracellular receptor domain, a transmembrane region, and an intracellular domain with tyrosine kinase function. It is a member of the ErbB family of receptors, a subfamily of four closely related receptor tyrosine kinases: EGFR (ErbB-1), HER2/neu (ErbB-2), HER3 (ErbB-3), and HER4 (ErbB-4) [1]. Upon binding of EGF or other ligands, EGFR is activated and induces the activation of downstream signaling pathways, including Ras-MAPK, PI3K/Akt, JAK/STAT, and PLCγ/PKC pathways, which leads to tumor cell proliferation, angiogenesis, tumor invasion, metastasis, and inhibition of apoptosis [2, 3]. EGFR is overexpressed in a wide variety of solid tumors and is associated with poor prognosis [4].

Several approaches have been developed that target the EGFR to interfere with EGFR-mediated cellular effects, preventing the growth of EGFR-expressing tumors [1, 5]. The two most extensively studied to date consist of monoclonal antibodies blocking EGFR binding sites on the extracellular domain of the receptor and small-molecule compounds inhibiting intracellular tyrosine kinase activity. Anti-EGFR mAbs have been widely used in mCRC, including cetuximab (Erbitux, IMC-C225) and panitumumab (Vectibix, ABX-EGF). EGFR tyrosine kinase inhibitors (EGFR-TKIs) are effective in treating EGFR-mutated lung cancer, whereas they have thus far shown little activity in colorectal cancer [6].

The development of panitumumab and cetuximab is a milestone in the history of mCRC treatment, significantly improving the PFS and OS [7], but anti-EGFR mAb therapy is only effective for some mCRC patients [8, 9]. *RAS* mutations have been demonstrated to be negative predictive biomarkers of anti-EGFR mAb response and survival benefit [10, 11]. Indeed, *KRAS* and *NRAS* mutations activate downstream pathways independently of EGFR status and induce primary drug resistance. However, many tumors containing wild-type *KRAS* and *NRAS* still do not respond to these therapies, suggesting that other molecular mechanisms of resistance exist.

Several retrospective evidence suggested that mutations of *BRAF*, *PIK3CA*, loss of *PTEN*, aberrant expression of EGFR and its ligands amphiregulin (AREG) and epiregulin (EREG), amplification or overexpression of *HER2/3*, and dysregulation of microRNAs could be prognostic or predictive biomarkers of anti-EGFR mAb in *RAS* wt mCRC patients [12–20]. However, most of the conclusions are still controversial [21–25]. To date, only *RAS* and *BRAF* mutations have been incorporated into routine clinical practice, and the role of other biomarkers still needs to be validated. *BRAF* oncogene mutations are strong prognostic markers, but the predictive value for anti-EGFR mAb therapy remains a matter of debate [26, 27].

Therefore, this systematic review pooled the prognostic and predictive value of these additional biomarkers to further select patients with *RAS* wt mCRC who are most likely to benefit from EGFR-targeted therapy.

## Methods

We performed this review according to the guidance of the Preferred Reported Items for Systematic Reviews and Meta-Analyses (PRISMA) 2020 statement [28]. The PRISMA compliance has been delineated in the PRISMA checklist table provided in Supplementary Table S1. A prospective protocol was registered in PROSPERO, CRD42022303340.

### Eligibility criteria

Criteria for considering studies included: 1) Types of studies, prospective randomized clinical trials, or prospective-retrospective biomarker analysis. 2) Types of participants, *RAS* wt mCRC; 3) types of interventions, matched anti-EGFR mAb therapy (either as monotherapy or in combination with standard‐of‐care palliative chemotherapy) versus alternative therapy; 4) Types of outcome measures, progression‐free survival (PFS, defined as the time from trial enrolment to a composite of disease progression and death), overall survival (OS, defined as the time from trial enrolment to death from any cause), and/or overall response rate (ORR, defined as the percentage of people who achieved either a complete response or partial response) [29].

Due to the disparity in the trial protocol designs and the executing clinical centers, there were differences in the number and sort of investigated biomarkers among these studies. When we analyzed the prognostic and predictive values of each biomarker, we selected those studies containing the required data of biomarkers, which showed different numbers of studies in every analysis.

### Search strategy

We systematically searched the Cochrane Library, PubMed, and Embase databases (up to 7 February 2022). The searches were rerun before the final analysis. The search strategy was presented in Additional file 1. All relevant articles were identified on PubMed to conduct a further search using the 'related articles' feature. In addition, we manually examined the citation lists of included studies and previous systematic reviews. Two authors (XL and YL) performed the search independently, in parallel.

### Study selection

We imported all records retrieved by electronic searching to Endnote 20 software and removed duplicates. Three authors (YL, XZ, and JS) examined the remaining references independently, in parallel. We excluded those studies that clearly do not meet the inclusion criteria and obtained the full text of potentially relevant references. Independently, three investigators assessed the eligibility of the retrieved studies. Any disagreement was resolved through discussion or, if required, consulted a fourth person (ZY). We identified and excluded duplicate reports and collated multiple reports of the same study so that each study, rather than each report, was the unit of interest in the review.

### Data extraction

Two authors (XZ, and JS) independently extracted data from included studies, resolving discrepancies by consensus or a third author (HF). Data were collected included the following: authors, publication years, journals, trial names, study design, participant demographics and characteristics, treatment protocols (lines of treatment and study treatment protocols), the status of biomarkers, outcomes (PFS, OS, and/or ORR), and results (numbers of events, hazard ratio (HR) and odds ratio (OR), and 95% confidence interval (CI). We extracted all information for data from the same trial presented in multiple publications and reported it as a single trial.

### Study risk of bias assessment

We assessed and reported the risk of bias for each included study according to the Newcastle–Ottawa Scale (NOS), which is categorized into three dimensions: selection of study groups; comparability of groups; and assessment of outcomes [30, 31]. The scale for cohort studies was used because nearly all included studies were based on retrospective biomarker analysis using archived tumor specimens, and biomarker status was not a matter of randomization. Risk of bias assessments was performed independently by two authors (XL and YL) and any differences were resolved by discussion.

### Statistical analysis

For prognostic and predictive analyses, PFS, OS, and ORR by treatment arm were assessed in subgroups of *RAS* wt patients according to the status of biomarkers. We used HRs with 95% CIs as the measure of effect for the time‐to‐event outcomes (PFS and OS). For the dichotomous outcome (ORR), OR with 95% CI was calculated. For crossover trials, we only used pre-crossover data for pooling to minimize potential bias from carry-over effects [32].

The prognostic value of biomarkers was investigated by comparing outcomes in *RAS* wt patients with different statuses of biomarkers using the HRs and ORs in the experimental and control arms, respectively. The predictive value of biomarkers was investigated by comparing the HRs or ORs of anti-EGFR mAb therapy (experimental arm) versus no anti-EGFR mAb therapy (control arm). Evidence for treatment effect modification by different statuses of biomarkers was evaluated by interaction tests. HRs were generally adjusted for covariates but varied to account for differences between studies. The ORs were not adjusted. The pooled HRs/ORs correspond to stratified Cox proportional hazards and logistic regression models, respectively. The HRs/ORs of interaction were pooled as proposed by Fisher et al. [33]. For data that could not be pooled statistically using meta‐analysis, we conducted a narrative synthesis of results adhering to the Synthesis Without Meta‐analysis (SWiM) guideline [34].

Heterogeneity between studies was evaluated by visual inspection of forest plots and quantified using the *I*^2^ statistic [35, 36]. *I*^2^ > 50% may represent substantial heterogeneity, in which case a random-effect (RE) model was used; otherwise, a fixed-effect (FE) model was used. The publication bias risk was assessed using funnel plots and Egger's linear regression test. Sensitivity analyses were performed to investigate the impact of excluding trials with a high risk of bias. Prespecified analyses were undertaken by grouping trials according to the anti-EGFR mAb therapy (cetuximab or panitumumab), the line of therapy, and treatment modalities in the control arm (with or without bevacizumab). All reported *P* values were two-sided, and all statistical analyses were carried out using R statistical software (version 4.1.2; with the meta_v5.2–0 packages).

## Results

### Overview of included studies and risk of bias assessment

**Fig. 1:**
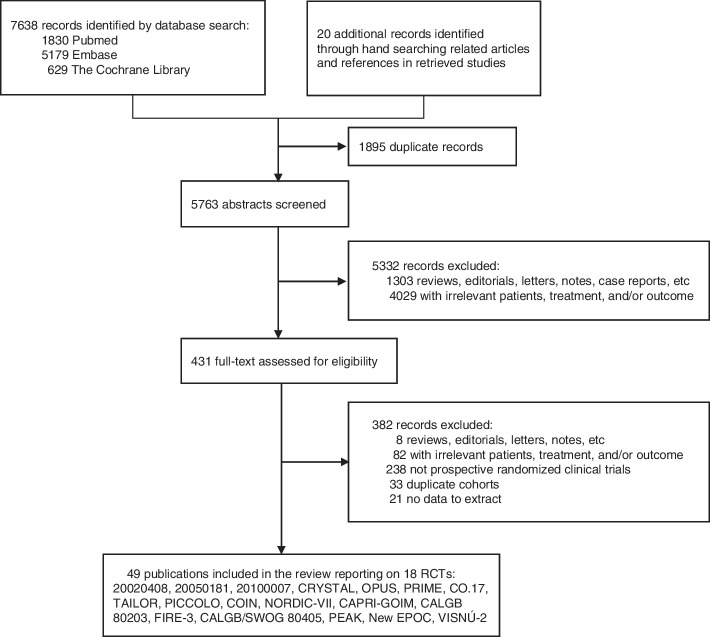
Flow chart of study selection

The search retrieved a total of 7658 articles that have been thoroughly reviewed for entry criteria (Fig. 1). Eighteen trials comprising 13,507 intention-to-treat (ITT) populations were finally identified that met the inclusion criteria (Table 1; Supplementary Table S2). Thirteen trials compared the addition of an anti-EGFR mAb with background treatment (FOLFIRI, FOLFOX, FLOX, irinotecan, oxaliplatin/irinotecan plus fluoropyrimidine, or best supportive care), and five compared the addition of an anti-EGFR mAb or bevacizumab to chemotherapy (FOLFOX or FOLFIRI). Twelve trials evaluated cetuximab and six assessed panitumumab. *RAS* mutation status was evaluable in 36%–100% of the ITT populations. Wild-type *RAS* accounts for approximately 59% of evaluable patients. 14 trials were available to assess the prognostic and predictive value of *BRAF* mutations by NGS, sanger sequencing, PCR and pyrosequencing, 4 trials for that of *PIK3CA* mutations by NGS, PCR and pyrosequencing, and 2 trials for that of non-functional PTEN by NGS and IHC. Three trials examined the combined effect of multiple biomarkers mutations. In addition, 8 trials assessed the value of EGFR and its ligands by IHC and PCR, 3 trials for that of other members of the HER family by PCR, and 3 trials for that of microRNA by PCR. The risk of bias assessments was summarized in Supplementary Table S3.

**Table 1:** Summary of trials included in the review

Trial name	Trial characteristics									
	**Phase of trial**	**Anti-EGFR mAb therapy**	**Treatment line**	**Background therapy**	**Bevacizumab in control arm?**	**ITT, *N***	***RAS* status,* N* (%) of ITT**		***RAS* wt, *N* (%)**	**Biomarker analysis**
**20020408** [15, 37]	III	Panitumumab	3rd	BSC	No	463	*KRAS* codons 12, 13, and 61	288 (62)	153 (53)	*BRAF*, *PIK3CA*, *PTEN*, *EGFR* GCN
**20050181** [38]	III	Panitumumab	2nd	FOLFIRI	No	1186	*KRAS* and *NRAS* exons 2, 3, 4	1014 (85)	421 (42)	*BRAF*
**20100007** [39]	III	Panitumumab	2nd	BSC	No	377	*KRAS* and *NRAS* exons 2, 3, 4	377 (100)	270 (72)	*BRAF*
**CRYSTAL** [40, 41]	III	Cetuximab	1st	FOLFIRI	No	1198	*KRAS* exon 2	1063 (89)	666 (63)	*BRAF*, EGFR
**OPUS** [40]	II	Cetuximab	1st	FOLFOX4	No	337	*KRAS* exon 2	315 (93)	179 (57)	*BRAF*
**PRIME** [10]	III	Panitumumab	1st	FOLFOX4	No	1183	*KRAS* and *NRAS* exons 2, 3, 4	1060 (90)	512 (48)	*BRAF*
**CO.17** [42, 43]	III	Cetuximab	2nd	BSC	No	572	*KRAS* exon 2	394 (69)	230 (58)	*BRAF*, *PIK3CA*, PTEN,* EREG*
**TAILOR** [44, 45]	III	Cetuximab	1st	FOLFOX4	No	393	*KRAS* and *NRAS* exons 2, 3, 4	393 (100)	393 (100)	EGFR
**PICCOLO** [46–51]	III	Panitumumab	2nd	Irinotecan	No	696	*KRAS* codons 12, 13, and 61	523 (75)	523 (100)	*BRAF*, *PIK3CA*, *KRAS/NRAS/BRAF/PIK3CA*, *EGFR* CN*,* EREG/AREG, EREG, AREG, *HER3*, *MiR 31-3p*
**COIN** [52–54]	III	Cetuximab	1st	Oxaliplatin and fluoropyrimidine	No	2445	*KRAS* codons 12, 13, and 61	1949 (80)	1125 (58)	*BRAF*, *PIK3CA*, EGFR*, EREG/AREG*
**NORDIC-VII** [55]	III	Cetuximab	1st	FLOX	No	566	*KRAS* and *NRAS* exons 2, 3, 4	457 (81)	247 (54)	*BRAF*
**CAPRI-GOIM** [56]	II	Cetuximab	2nd	FOLFOX	No	153	*KRAS* exon 2	153 (100)	153 (100)	*KRAS/NRAS/BRAF/PIK3CA*
**CALGB 80203** [57]	II	Cetuximab	1st	FOLFOX or FOLFIRI	No	238	*KRAS* exon 2	103 (43)	55 (53)	*EGFR, EREG, AREG, HER2, HER3*, *HER4*
**FIRE-3** [58, 59]	III	Cetuximab	1st	FOLFIRI	Yes	592	*KRAS* and *NRAS* exons 2, 3, 4	515 (87)	343 (67)	*BRAF*, *MiR 31-3p*, *MiR-21*
**CALGB/SWOG 80405** [60]	III	Cetuximab	1st	mFOLFOX6 or FOLFIRI	Yes	2326	*KRAS* exon 2	843 (36)	600 (71)	*BRAF*
**PEAK** [61]	II	Panitumumab	1st	mFOLFOX6	Yes	285	*KRAS* and *NRAS* exons 2, 3, 4	250 (88)	170 (68)	*BRAF*
**New EPOC** [62, 63]	III	Cetuximab	1st	Oxaliplatin/irinotecan plus fluorouracil	Yes	257	*KRAS* codons 12, 13, and 61	257 (100)	257 (100)	*MiR 31-3p*
**VISNÚ-2** [64]	II	Cetuximab	1st	FOLFIRI	Yes	240	*KRAS* exons 2 and 3	240 (100)	240 (100)	*BRAF/ PIK3CA*

### Markers downstream of EGFR

#### BRAF Mutations

##### Prognostic role of BRAF Mutations

**Fig. 2:**
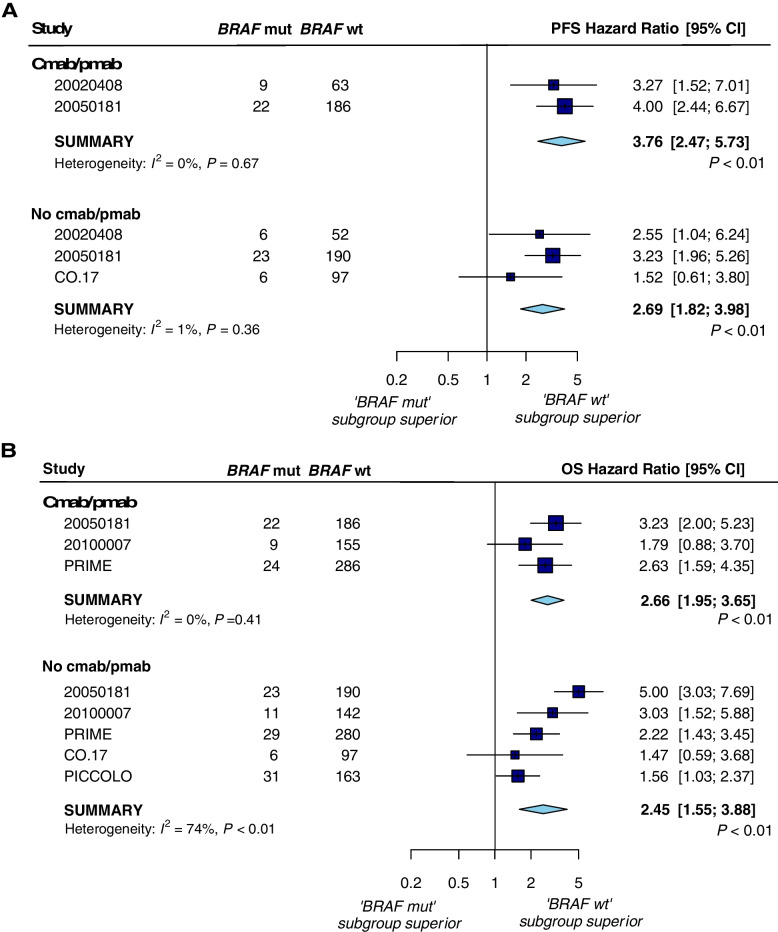
Forest plots for the prognostic analyses of *BRAF* mutations in the control and experimental arms for progression-free survival **a** and overall survival **b**. Cmab, cetuximab; Pmab, panitumumab; mut, mutant; wt, wild-type. OS, overall survival; PFS, progression-free survival

Six trials (five panitumumab trials and one cetuximab trial) reported PFS or OS data that could be used to assess the prognostic value of *BRAF* mutations. For the anti-EGFR therapy arm, pooled analyses (Fig. 2a, b) showed an overall HR of 3.76 [2.47–5.73] (*P* < 0.01) for PFS and 2.66 [1.95–3.65] (*P* < 0.01) for OS in the absence of any heterogeneity between trials, indicating a negative prognostic effect of *BRAF* mutation. Results were similar in the control arm, with an overall HR for PFS of 2.69 [1.82–3.98] (*P* < 0.01; heterogeneity test *P* = 0.36, *I*^2^ = 1%) but less pronounced than in the experimental arm. The overall HR for OS in the control arm was 2.45 [1.55–3.88] (*P* < 0.01), but there was substantial heterogeneity (*P* < 0.01; *I*^2^ = 74%). Sensitivity analysis showed that after excluding 20,050,181 study, there was no longer significant heterogeneity for OS (*P* = 0.33; *I*^2^ = 12%) with an overall HR of 1.95[1.50–2.54] (*P* < 0.01).

##### Predictive role of BRAF Mutations

**Fig. 3:**
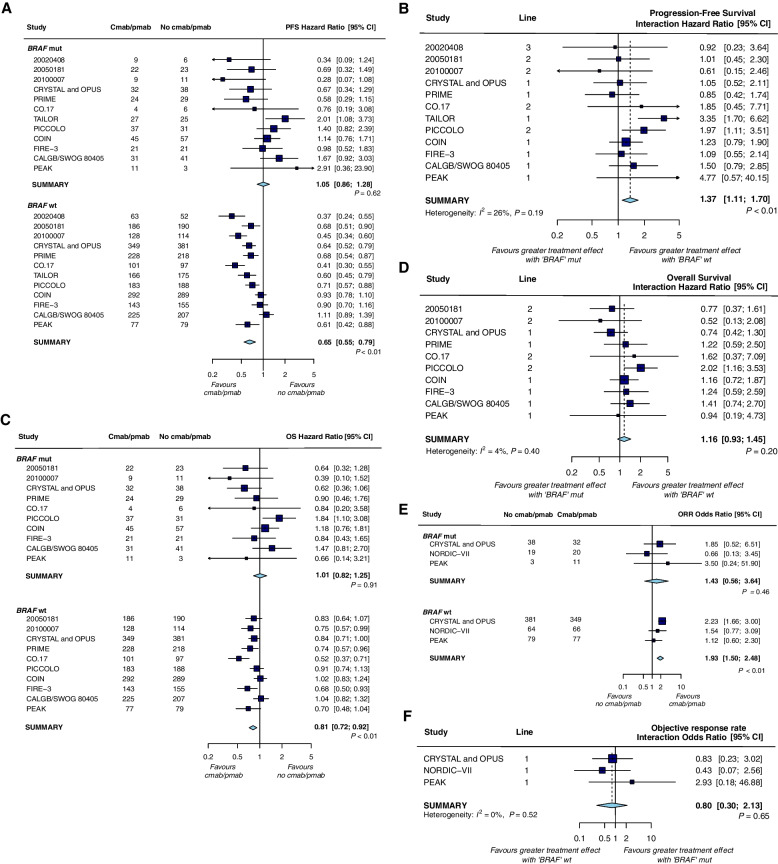
Forest plots for predictive analyses of *BRAF* mutations in trials comparing experimental arm with control arm—**a**, **b** progression-free survival, **c**, **d** overall survival and **e**, **f** objective response rate. PFS, progression-free survival; OS overall survival; ORR, objective response rate

Thirteen trials reported sufficient data to assess whether the efficacy of anti-EGFR mAb differed between *BRAF* subgroups. PFS data were available for all 13 RCTs. Overall, the addition of anti-EGFR mAb did not increase PFS in patients with *BRAF* mutant tumors compared with controls [HRs of 1.05 (0.86–1.28); *P* = 0.62]; whereas a significant benefit of anti-EGFR mAb therapy was observed in patients with *BRAF* wt tumors [HRs of 0.65 (0.55–0.79); *P* < 0.01] (Fig. 3a). OS data were available from 11 trials except the 20,020,408 and TAILOR trials. Based on the pharmacogenomic substudies of 11 RCTs, the hazard ratio for OS benefit with anti-EGFR mAb therapy was 1.01 (0.82–1.25) for *BRAF* mutant tumors as compared with 0.81 (0.72–0.92) for *BRAF* wt tumors (Fig. 3c). Similar patterns were observed for ORR data obtained from 4 trials, with a trend toward greater benefit in the anti-EGFR mAb therapy arm in patients with *BRAF* wt tumors [OR = 1.93 (1.50, 2.48); *P* < 0.01] compared with patients with *BRAF* mutant tumors [OR = 1.43 (0.56, 3.64); *P* = 0.46] (Fig. 3e).

Treatment interaction tests showed a significant difference in the PFS benefit of anti-EGFR mAb therapy between *BRAF* mutant and *BRAF* wt tumors (HR = 1.37 (1.11, 1.70), interaction test *P* < 0.01) (Fig. 3b). However, there was little difference was observed between *BRAF* mutant and *BRAF* wt tumors with respect to either OS efficacy (HR = 1.16 (0.93, 1.45), interaction test *P* = 0.40) (Fig. 3d) or ORR efficacy (OR = 0.80 (0.30, 2.13), interaction test *P* = 0.65) (Fig. 3f). No significant inter heterogeneity was evident between studies for the interaction test between *BRAF* subgroups(*I*^2^ of PFS, OS, and ORR were 26%, 4%, and 0%, respectively.). Visual inspection and linear regression tests of funnel plot asymmetry did not indicate significant publication bias.

In terms of PFS, the predictive role of *BRAF* status was not significantly different between studies with different anti-EGFR mAb agents (*P* for interaction = 0.55), different lines of therapy (*P* for interaction = 0.88), and with or without bevacizumab in the control arm (*P* for interaction = 1.00) (Table 2). However, the predictive effect was significant for studies with cetuximab, first-line therapy, or without bevacizumab (*P* values < 0.01, 0.02, and < 0.01, respectively) but not for studies with panitumumab, second-line and above treatment, or with bevacizumab (*P* values 0.21, 0.10 and 0.18, respectively). Regarding OS and ORR, no predictive effect was observed after stratification for anti-EGFR mAb used, line of treatment, and whether or not bevacizumab was included in the control arm.

**Table 2:** Relative size of anti-EGFR mAb efficacy for *BRAF* mutant tumors compared to *BRAF* wild-type tumors. Results stratified by anti-EGFR drug, line of therapy, and bevacizumab in control arm

**Subgroup**	**PFS**			*P* value for interaction*	**OS**			*P* value for interaction^*^		**ORR**		*P* value for interaction^*^
	**N Trials**	**Interaction Hazard Ratio (95% CI)**	**Interaction *P* value**		**N Trials**	**Interaction Hazard Ratio (95% CI)**	**Interaction *P* value**		**N Trials**	**Interaction Odds Ratio (95% CI)**	**Interaction *P* value**	
**Anti-EGFR drug**				0.55				0.56				0.33
Cetuximab	7	1.44 (1.11, 1.87)	< 0.01		6	1.10 (0.82, 1.46)	0.52		3	0.66 (0.23, 1.89)	0.44	
Panitumumab	6	1.26 (0.88, 1.80)	0.21		5	1.26 (0.88, 1.79)	0.21		1	2.93 (0.18, 46.88)	0.45	
**Line of therapy**				0.88				0.88				
First	8	1.36 (1.06, 1.74)	0.02		7	1.10 (0.84, 1.43)	0.50		3	0.80 (0.30, 2.13)	0.65	-
≥ Second	5	1.41 (0.94, 2.11)	0.10		4	1.16 (0.60, 2.25)	0.65		0	-		
**Bevacizumab in control arm?**				1.00				0.60				0.33
Yes	3	1.37 (0.87, 2.16)	0.18		3	1.29 (0.81, 2.06)	0.28		3	0.66 (0.23, 1.89)	0.44	
No	10	1.37 (1.08, 1.75)	< 0.01		8	1.12 (0.87, 1.44)	0.38		1	2.93 (0.18, 46.88)	0.45	

^*^Test comparing the HRs between trial subgroups (cetuximab; panitumumab; line of therapy; with/without bevacizumab;)

#### PIK3CA Mutations

##### Prognostic role of PIK3CA Mutations

Three trials analyzed the potential prognostic value of *PIK3CA* mutations, but only OS data based on two trials were available for pooling. In the control arm according to *PIK3CA* status (mutant versus wild-type), the HR for PFS was 1.10 [0.72–1.68] (*P* = 0.66) for the CO.17 trial, and the pooled HR for OS was 1.11 [0.80–1.55] (*P* = 0.54; heterogeneity test *P* = 1.00, *I*^2^ = 0%) for CO.17 and PICCOLO trials. The COIN trial also showed that regardless of the treatment arm, *PIK3CA* mutations did not affect PFS [HR = 1.06 (0.89–1.26); *P* = 0.49] or OS [HR = 0.91 (0.75–1.11); *P* = 0.37]. This was independent of whether the *PIK3CA* mutation was divided into mutations in exon 9 and exon 20. Based on the small amount of trial data available, no prognostic value of *PIK3CA* mutations was found for patients with mCRC.

##### Predictive role of PIK3CA Mutations

**Fig. 4:**
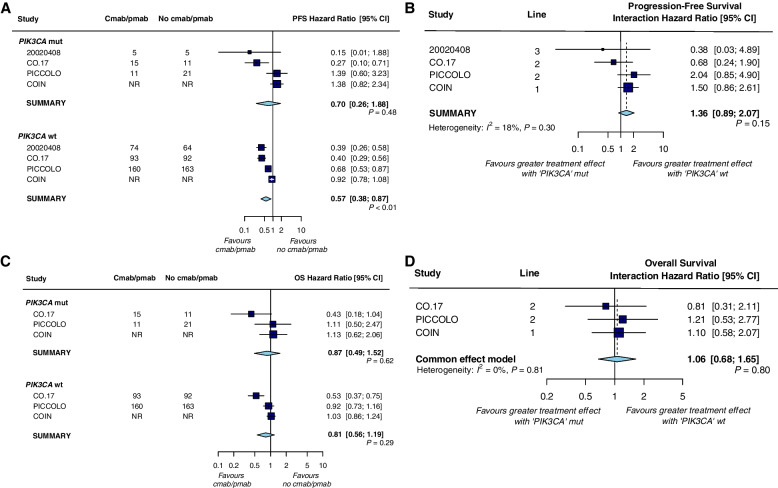
Forest plots for predictive analyses of *PIK3CA* mutations in trials comparing experimental arm with control arm—**a**, **b** progression-free survival and **c**, **d** overall survival. PFS, progression-free survival; OS overall survival

Analysis of data based on four trials indicated that for PFS (Fig. 4a), a significant benefit of anti-EGFR mAb therapy was observed in patients with *PIK3CA* wt tumors [HR = 0.57 (0.38–0.87); *P* < 0.01], whereas no benefit was observed in patients with *PIK3CA* mutant tumors [HR = 0.70 (0.26–1.88); *P* = 0.48]; for OS (Fig. 4c), no benefit was shown in patients with both wild-type and mutant *PIK3CA* tumors [HRs 0.81 (0.56–1.19); *P* = 0.29 and 0.87 (0.49–1.52); *P* = 0.62, respectively]. Treatment interaction tests between *PIK3CA* subgroups showed no difference in the predictive value of anti-EGFR mAb therapy for both PFS (HR = 1.36 (0.89, 2.07), interaction test *P* = 0.15; heterogeneity test *P* = 0.30, *I*^2^ = 18%, Fig. 4b) and OS (HR = 1.06 (0.68, 1.65), interaction test *P* = 0.80; heterogeneity test *P* = 0.81, *I*^2^ = 0% Fig. 4d) in the absence of significant heterogeneity between studies. Stratified analysis according to the type of anti-EGFR mAb and the line of therapy also did not observe any predictive effect regarding PFS and OS (Table 3).

**Table 3:** Relative size of anti-EGFR mAb efficacy for *PIK3CA* mutant tumors compared to *PIK3CA* wild-type tumors. Results stratified by anti-EGFR drug and line of therapy

**Subgroup**	**PFS**			*P* value for interaction^*^	**OS**			*P* value for interaction^*^
	**N Trials**	**Interaction Hazard Ratio (95% CI)**	**Interaction** ***P* value**		**N Trials**	**Interaction Hazard Ratio (95% CI)**	**Interaction** ***P* value**	
**Anti-EGFR drug**				0.53				0.70
Cetuximab	2	1.26 (0.77, 2.04)	0.36		2	1.00 (0.59, 1.70)	0.99	
Panitumumab	2	1.71 (0.75, 3.92)	0.21		1	1.21 (0.53, 2.77)	0.66	
**Line of therapy**				0.59				0.87
First	1	1.50 (0.86, 2.60)	0.15		1	1.10 (0.58, 2.06)	0.77	
≥ Second	3	1.19 (0.62, 2.26)	0.60		2	1.02 (0.55, 1.91)	0.95	

^*^Test comparing the HRs between trial subgroups (cetuximab; panitumumab; line of therapy)

#### Prognostic and Predictive role of Non-functional PTEN

Here we only analyzed the data descriptively because only two trials assessed the role of *PTEN* mutation and reduced PTEN expression in patients with *RAS* wt tumors, respectively. No quantitative analysis could not be performed. For the 20,020,408 trial, a favorable effect of panitumumab on PFS was observed in patients with *PTEN* wt tumors [n = 135; HR = 0.36 (0.25–0.52); *P* < 0.001] compared with no significant benefit in patients with *PTEN* mutant tumors [*n* = 9; HR = 0.11 (0.01–1.52); *P* = 0.10]. The interaction term did not suggest statistical significance (HR 0.31, interaction test *P* = 0.36). ORRs for mutant versus wild-type *PTEN* among patients with wild-type *KRAS* who were randomized to panitumumab were 14% (95% CI, 0–0.58) and 13% (95% CI, 0.06–0.22), respectively. No patients responded to BSC alone.

For the effect of PTEN expression deficiency, the CO.17 trial showed an HR of 0.99 (*P* = 0.98) for PFS and 1.13 (*P* = 0.70) for OS between PTEN-negative and positive subgroups in the control arm, indicating no prognostic significance. Regarding predictive value, the HRs for PFS and OS between cetuximab and best supportive care were 0.66 [0.31–1.41] and 0.66 [0.29–1.52], respectively, in patients with PTEN-positive tumors and 0.34 [0.20–0.57] and 0.63 [0.38–1.03], respectively, in patients with PTEN-negative tumors. Treatment interaction tests showed no significant association between PTEN status and the survival benefit of cetuximab therapy (HR 0.52, interaction test *P* = 0.16 for PFS; HR 0.95, interaction test *P* = 0.92 for OS). The ORR to cetuximab was 21% versus 15% in patients with *PTEN* positive versus negative tumors. These data suggest that in patients with *RAS* wt tumors, *PTEN* status was neither prognostic nor predictive of benefit from cetuximab.

#### Prognostic and predictive role of multiple biomarkers

Three trials evaluated the combined impact of multiple biomarkers on clinical outcomes of anti-EGFR therapy in mCRC. The role of *BRAF* and *PIK3CA* mutations in patients with *RAS* wt was analyzed in the VISNÚ-2 trial, which showed similar trends in PFS improvement in patients with *BRAF*/*PIK3CA* wt versus *BRAF*- and/or *PIK3CA*-mutated tumors in both treatment arms [FOLFIRI plus cetuximab: HR = 1.55 (0.83–2.89); *P* = 0.17; FOLFIRI plus bevacizumab: HR = 1.07 (0.61- 1.88); *P* = 0.83]. The predictive effect of chemotherapy plus EGFR antibody therapy compared with chemotherapy plus bevacizumab was not significantly different for patients with *BRAF*/*PIK3CA* wt and *BRAF*- or *PIK3CA*-mutated tumors both for PFS (HR 1.12, interaction test *P* = 0.79), OS (HR 0.87, interaction test *P* = 0.73), and ORR (OR 0.71, interaction test *P* = 0.65).

**Fig. 5:**
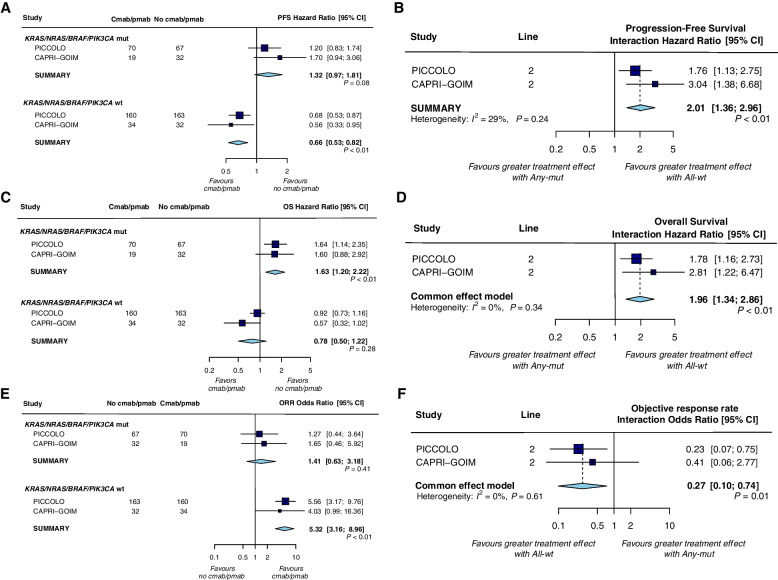
Forest plots for predictive analyses of multiple biomarkers in trials comparing experimental arm with control arm—**a**, **b** progression-free survival, **c**, **d** overall survival and **e**, **f** objective response rate. PFS, progression-free survival; OS overall survival; ORR, objective response rate

Two additional trials assessed whether the efficacy of anti-EGFR mAb differed between *KRAS*, *NRAS*, *BRAF,* and *PIK3CA* combined biomarker subgroups. The pooled analyses showed that significant PFS and ORR benefits of anti-EGFR mAb therapy were observed in patients with all-wt tumors [HR 0.66 (0.53–0.82); *P* < 0.01, OR 5.32 (3.16–8.96); *P* < 0.01] compared with no benefit in those with mutant (any mutation in *KRAS*/*NRAS*/*BRAF*/*PIK3CA* genes) tumors [HR 1.32 (0.97–1.81); *P* = 0.08, OR 1.41 (0.63–3.18); *P* = 0.41] (Fig. 5a, e). The results for OS showed a similar trend (Fig. 5c), with significantly shorter OS in patients with any mutant tumors [HR 1.63 (1.20, 2.22); *P* < 0.01] compared to patients with all-wild type tumors [HR 0.78 (0.50, 1.22); *P* = 0.28]. Treatment interaction tests showed that the predictive value of anti-EGFR mAb therapy was significantly different for patients with mutant and wild-type *KRAS*/*NRAS*/*BRAF*/*PIK3CA* tumors all for PFS (HR = 2.01 (1.36, 2.96), interaction test *P* < 0.01; heterogeneity test *P* = 0.24, *I*^2^ = 29%, Fig. 5b), OS (HR = 1.96 (1.34, 2.86), interaction test* P* < 0.01; heterogeneity test *P* = 0.34, *I*^2^ = 0%, Fig. 5d) and ORR (OR = 0.27 (0.10, 0.74), interaction test* P* = 0.01; heterogeneity test *P* = 0.61, *I*^2^ = 0%, Fig. 5f).

### EGFR and its ligands

#### Prognostic and predictive role of EGFR

**Fig. 6:**
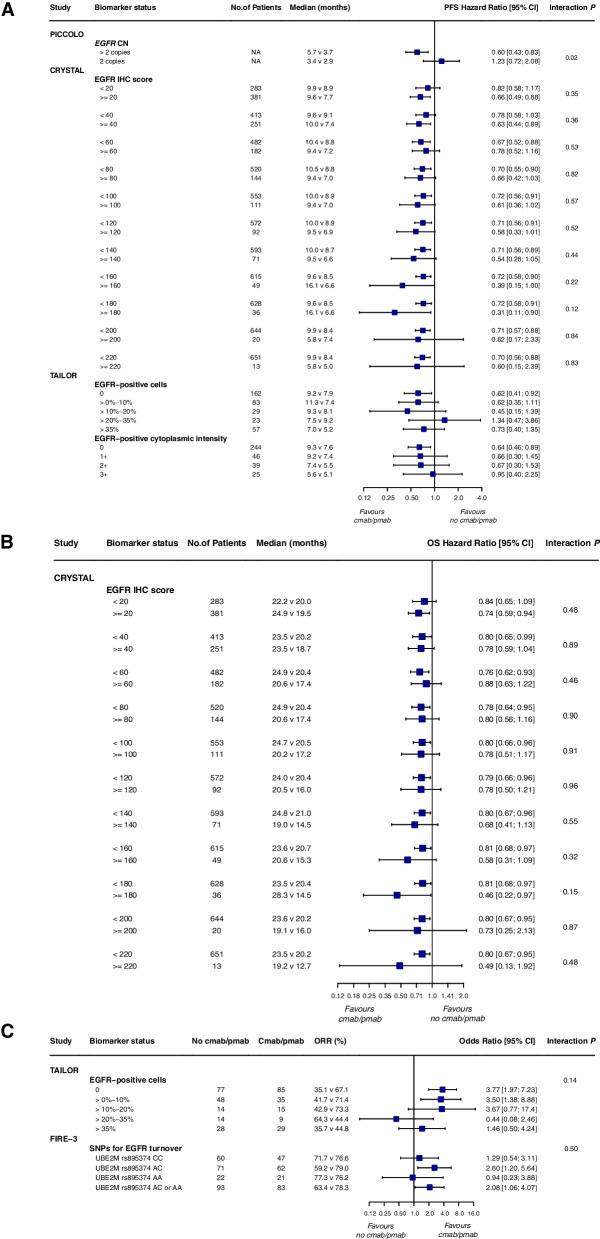
Forest plots for predictive analyses of EGFR in trials comparing experimental arm with control arm—**a** progression-free survival, **b** overall survival and **c** objective response rate. NA, not available; PFS, progression-free survival; OS overall survival; ORR, objective response rate

Because of the significant heterogeneity of EGFR status, detection methods, and determination criteria in different studies, no quantitative synthesis of data was performed. Three trials used immunohistochemistry to assess the correlation between EGFR immunostaining and clinical response to anti-EGFR mAb therapy. The COIN trial showed a prognostic effect of EGFR on PFS at the standardized cutoff point of < 10% vs. ≥ 10% [HR 1.25 (1.05–1.50);* P* = 0.015] in the *KRAS* wt cohort (Table 4). There was no evidence of EGFR immunohistochemistry's predictive role, regardless of the cutoff point (data not shown). Data from the CRYSTAL and TAILOR studies also confirm that adding cetuximab to chemotherapy improved the survival benefit of first-line treatment for patients with *RAS* wt mCRC, irrespective of tumor EGFR status (Fig. 6a-c).

**Table 4:** Effect of EGFR for *RAS* wild-type patients, according to treatment

Study	Biomarker	Subgroup	N	PFS			OS			ORR		
				**Median, months**	**HR (95% CI)**	***P* value**	**Median, months**	**HR (95% CI)**	***P* value**	**Rate, %**	**Odds ratio (95% CI)**	***P* value**
**COIN**												
	**EGFR membrane staining cells**	**Cetuximab ± oxaliplatin and fluoropyrimidine (*N* = NR)**			1.25 (1.05–1.50)	0.015		NR	NR		NR	NR
		< 10%	NR	NR			NR			NR		
		≥ 10%	NR	NR			NR			NR		
**CALGB 80203**												
	***EGFR* mRNA**^**a**^	**Cetuximab + FOLFOX or FOLFIRI (N = 26)**			0.77 (0.50–1.21)	NR		1.07 (0.77–1.46)	NR		NR	NR
		high	NR	NR			NR			NR		
		low	NR	NR			NR			NR		
		**FOLFOX or FOLFIRI (*N* = 29)**			1.04 (0.63–1.73)	NR		1.21 (0.69–2.11)	NR		NR	NR
		high	NR	NR			NR			NR		
		low	NR	NR			NR			NR		
**20,020,408 and 194**^**b**^												
	***EGFR* GCN**	**Panitumumab + BSC (*N* = 58)**			NR	0.039		NR	0.015		NR	0.0009^c^
		≥ 2.5 copies/nucleus	19	NR			NR			30		
		< 2.5 copies/nucleus	39	NR			NR			0		
		**BSC (*N* = 34)**				0.635		NR	NR		NR	NR
		≥ 2.5 copies/nucleus	8	NR			NR			NR		
		< 2.5 copies/nucleus	26	NR			NR			NR		
	***EGFR* chromosome 7 polysomy**	**Panitumumab + BSC (*N* = 58)**			NR	0.029		NR	0.014	31.6	NR	0.0007^c^
		≥ 40%	22	NR			NR					
		< 40%	36	NR			NR			0		
**PICCOLO**												
	***EGFR* CN**	**Panitumumab + Irinotecan (*N* = NR)**			NR	NR			NR		NR	0.01
		> 2 copies	NR	NR			NR	NR		45.3		
		2 copies	NR	NR			NR	NR		18.7		
		**Irinotecan (*N* = NR)**			NR	0.98		NR	0.97		NR	1.0
		> 2 copies	NR	NR			NR			13.3		
		2 copies	NR	NR			NR			12.9		

^a^where expression level is dichotomized at the median as "high" or "low". ^b^Patients in the BSC arm of the 408 study who had disease progression could enroll in the panitumumab open-label continuation 194 study (Clinicaltrials.gov, NCT00113776). ^c^EGFR GCN ≥ 2.47 vs < 2.47; Chromosome 7 polysomy ≥ 43% vs < 43%

The CALGB 80203 trial reported data from patients with EGFR expression evaluated by quantitative polymerase chain reaction and no prognostic association was found between this parameter and survival outcomes in *KRAS* wt patients [HRs of 0.80 (0.58–1.09); *P* = 0.168 and 1.04 (0.81–1.34); *P* = 0.748 for PFS and OS, respectively]. EGFR gene expression levels were also not a potential predictive marker for cetuximab benefit (HR 0.74, interaction test *P* = 0.37 for PFS; and HR 0.88, interaction test *P* = 0.86 for OS).

Two trials (the 20,020,408 and PICCOLO trials) provided data from *EGFR* amplification patients, showing that patients with increased *EGFR* gene copy number had higher response rates and longer PFS to anti-EGFR mAb compared to patients with normal *EGFR* gene copy number (Table 4; Fig. 6a). Data from the 20,020,408 trial showed that in patients treated with panitumumab, the mean *EGFR* GCN ≥ 2.5/nucleus or percentage of chromosome 7 polysomy ≥ 40% predicted better PFS (*P* = 0.039 and 0.029, respectively) and OS (*P* = 0.015 and 0.014, respectively). Six of 20 patients with *EGFR* GCN ≥ 2.47/nucleus and six of 19 patients with chromosome 7 polysomy ≥ 43% achieved an objective response ((*P* = 0.0009 and 0.0007, respectively). In contrast, no patients had tumor response when the *EGFR* GCN or chromosome 7 polysomy was less than this value. In this trial, there was no correlation between *EGFR* GCN and chromosome 7 polysomy status and PFS in patients receiving supportive care, suggesting that this parameter is not prognostic in mCRC. The PICCOLO trial showed a similar trend, with no prognostic effect of EGFR copy number gain on PFS (*P* = 0.98) and OS (*P* = 0.97). However, it was predictive of panitumumab benefit, with median PFS of 5.7 vs 3.7 months in *RAS* wt patients with EGFR-gain [HR 0.60 (0.43–0.83), *P* = 0.002], but no benefit in patients with normal EGFR [3.4 vs 2.9 months, HR 1.23 (0.72–2.08); *P* = 0.45) (HR 0.49, interaction test *P* = 0.02). In *RAS* wt patients, EGFR gain was associated with higher response rates than normal in the irinotecan plus panitumumab arm (45.3% vs. 18.7%, *P* = 0.01) but not in the irinotecan arm (13.3% vs. 12.9%, *P* = 1.0) (Table 4). The interaction was not significant (*P* = 0.22).

#### Prognostic and Predictive role of EGFR Ligands

Five trials evaluated the EGFR ligands EREG and AREG as prognostic and predictive biomarkers. We did not quantitatively synthesize the data due to the apparent differences between studies.

**Fig. 7:**
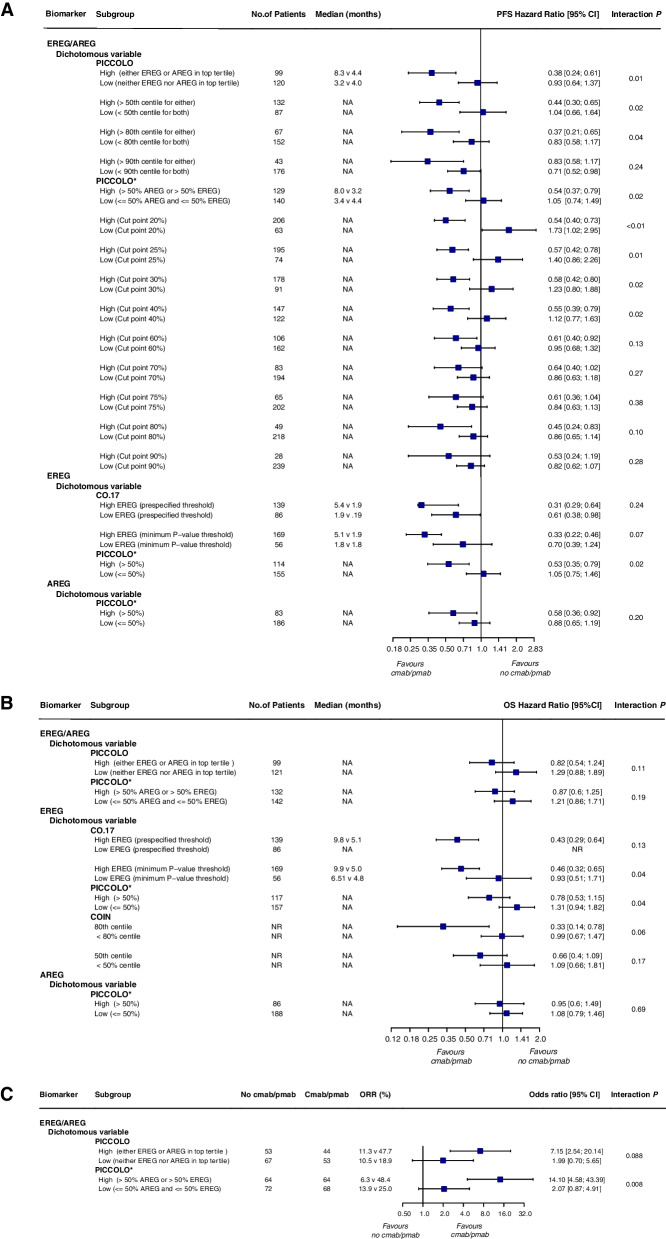
Forest plots for predictive analyses of EGFR ligands in trials comparing experimental arm with control arm—**a** progression-free survival, **b** overall survival and **c** objective response rate. NA, not available; PFS, progression-free survival; OS overall survival; ORR, objective response rate

In terms of EREG/AREG as a combined dichotomous biomarker, data from the PICCOLO trial confirmed that high ligand mRNA levels or IHC positivity are predictive biomarkers of benefit from panitumumab treatment in patients with metastatic colorectal cancer (Fig. 7a-c). In *RAS* wt patients with high ligand mRNA levels (either ligand in the top tertile), panitumumab treatment had a significantly longer PFS compared with control treatment [HR 0.38 (0.24–0.61); *P* < 0.001]. However, there was no benefit of panitumumab in *RAS* wt patients with low ligand mRNA levels (neither ligand in top tertile) [HR, 0.93 (0.64–1.37); *P* = 0.73]. The ligand-treatment interaction was significant (HR 0.41, interaction test *P* = 0.01). The effects on OS (HR 0.64, interaction test *P* = 0.11) and ORR (OR 3.69, interaction test *P* = 0.088) were less significant. The trial also explored several alternative cutpoints, including the 50th, 80th, and 90th centiles, but none separated the beneficiary/non-beneficiary population to a greater extent. Analysis of immunohistochemistry showed similar results, with high ligand IHC positivity (> 50% AREG or > 50% EREG) associated with significant PFS and ORR benefit with panitumumab [HR for PFS 0.54 (0.37–0.79); *P* = 0.001 and OR for ORR 14.1 (4.58, 43.39)); *P* = 0.000]; and no benefit in patients with low ligand IHC positivity (≤ 50% AREG and ≤ 50% EREG) [HR for PFS 1.05 (95% CI, 0.74–1.49); *P* = 0.78, and OR for ORR 2.07 (0.87, 4.91);* P* = 0.10]. Treatment interaction tests were significant both for PFS (HR 0.51, interaction test *P* = 0.02) and ORR (OR 6.81, interaction test *P* = 0.008). The results for OS were less significant (HR 0.72, interaction test *P* = 0.19). The effects of different cutoffs were also examined here and found that interactions remained significant across the 20% to 50% cutoff range. For the prognostic role of tumor EREG/AREG expression in *RAS* wt patients, no prognostic effect of high versus low expresser status on PFS or OS was seen in the subgroup treated with irinotecan alone (Table 5).

**Table 5:** Effect of EGFR Ligands for *RAS* wild-type patients, according to treatment

Biomarker	Study	Subgroup	N	PFS				OS			
				**Median, months**	**HR (95% CI)**	***P* value**	**Interaction *P***	**Median, months**	**HR (95% CI)**	***P* value**	**Interaction *P***
**EREG/AREG**											
	**PICCOLO**	**Irinotecan (*N* = NR)**			1.03 (0.70, 1.50)	0.89			0.81 (0.55, 1.19)	0.29	
		High (either EREG or AREG in top tertile)	NR					NR			
		Low (neither EREG nor AREG in top tertile)	NR	NR				NR			
	PICCOLO^*^	**Irinotecan (*N* = 140)**			1.11 (0.77, 1.62)	0.57			0.93 (0.65, 1.34)	0.70	
		High (> 50% AREG or > 50% EREG)	66	NR				NR			
		Low (≤ 50% AREG and ≤ 50% EREG)	74	NR				NR			
**EREG**											
	**CO.17**	**BSC (*N* = 111)**			0.80 (0.59, 1.09)	0.16			0.82 (0.58, 1.15)	0.24	
		High (prespecified threshold (ΔCt = 6.27))	73	NR				NR			
		Low (prespecified threshold (ΔCt = 6.27))	38	NR				NR			
	**CALGB 80203**	**Cetuximab + FOLFOX or FOLFIRI (*N* = 26)**			0.84 (0.67, 1.00)	NR	0.41		0.91 (0.74, 1.08)	NR	0.70
		High (≥ the median)	NR	NR				NR			
		Low (< the median)	NR	NR				NR			
		**FOLFOX or FOLFIRI (*N* = 29)**			0.87 (0.71, 1.05)	NR			0.79 (0.66, 0.94)	NR	
		High (≥ the median)	NR	NR				NR			
		Low (< the median)	NR	NR				NR			
	**CO.17**	**Cetuximab + BSC (*N* = 114)**			0.88 (0.99, 0.79)	0.03	0.08		0.88 (0.99, 0.79)	0.04	0.30
		Continuous variable	114	NR				NR			
		**BSC (*N* = 111)**			NR	0.48			0.85 (0.96, 0.76)	0.01	
		Continuous variable	111	NR				NR			
	**PICCOLO**	**Panitumumab + Irinotecan (*N* = NR)**			0.85 (0.79, 0.93)	0.0003	0.08		0.86 (0.79, 0.93)	0.0005	0.72
		Log EREG	NR	NR				NR			
		**Irinotecan (*N* = NR)**			0.95 (0.86, 1.04)	0.27			0.87 (0.79, 0.95)	0.002	
		Log EREG	NR	NR				NR			
	PICCOLO^*^	**Panitumumab + Irinotecan (*N* = 134)**			0.93 (0.87, 0.98)	0.009	0.01		0.91 (0.86, 0.96)	0.001	0.17
		Continuous variable	134	NR				NR			
		**Irinotecan (*N* = 140)**			1.02 (0.96, 1.07)	0.57			0.96 (0.91, 1.02)	0.19	
		Continuous variable	140	NR				NR			
**AREG**											
	**CALGB 80203**	**Cetuximab + FOLFOX or FOLFIRI (*N* = 26)**			0.88 (0.67, 1.18)	NR	0.84		0.96 (0.70, 1.30)	NR	0.66
		High (≥ the median)	NR	NR				NR			
		Low (< the median)	NR	NR				NR			
		**FOLFOX or FOLFIRI (*N* = 29)**			0.87 (0.71, 1.07)	NR			0.84 (0.68, 1.03)	NR	
		High (≥ the median)	NR	NR				NR			
		Low (< the median)	NR	NR				NR			
	**PICCOLO**	**Panitumumab + Irinotecan (*N* = NR)**			0.79 (0.72, 0.87)	< 0.0001	0.008		0.85 (0.77, 0.93)	0.0006	0.07
		Log AREG	NR	NR				NR			
		**Irinotecan (*N* = NR)**			0.96 (0.85, 1.07)	0.47			0.95 (0.85, 1.06)	0.37	
		Log AREG	NR	NR				NR			
	PICCOLO*	**Panitumumab + Irinotecan (*N* = 134)**			0.95 (0.89, 1.02)	0.18	0.06		0.94 (0.88, 1.01)	0.09	0.43
		Continuous variable	134	NR				NR			
		**Irinotecan (*N* = 140)**			1.04 (0.97, 1.10)	0.27			0.98 (0.92, 1.05)	0.53	
		Continuous variable	140	NR				NR			

^*^Ligands expression was analyzed by immunohistochemistry

The trials also examined EREG and AREG separately as independent biomarkers. For EREG, contradictory results were presented between these studies. When examined as a dichotomous variable, in the prognostic analysis, *EREG* expression had a favorable prognostic effect on OS in the control arm [HR 0.79 (0.66–0.94)] in the CALGB 80203 trial but failed to show significance in the CO.17 and PICCOLO trials (Table 5). In predictive analysis, the CO.17 trial showed that patients with high *EREG* expression appeared to benefit more from cetuximab treatment than those with low expression (HR 0.49, interaction test *P* = 0.04 for OS, Fig. 7b), and the PICCOLO trial also found that the percentage of EREG IHC positivity > 50% predicted PFS (HR 0.50, interaction test *P* = 0.02, Fig. 7a) and OS (HR 0.60, interaction test *P* = 0.04, Fig. 7b) benefits of panitumumab. However, no predictive value was observed in the CALGB 80203 and COIN trials. As a continuous variable, a prognostic impact of *EGFR* expression on OS was observed in both the CO.17 and PICCOLO trials [HR 0.85 (0.96–0.76); *P* = 0.01 and 0.87 (0.80–0.94); *P* = 0.001, respectively] (Table 5), but did not predict survival benefit of anti-EGFR mAb therapy. In contrast, in the immunohistochemical analysis of the PICCOLO trial, EGFR IHC positivity was not prognostic for either PFS or OS in patients treated with irinotecan only but predicted a PFS benefit with panitumumab (HR 0.91, interaction test *P* = 0.01, Table 5). EREG status may have certain prognostic and predictive roles, but no definitive conclusions can be drawn based on current evidence.

When AREG was analyzed separately as a dichotomous variable, the CALGB 80203 and PICCOLO trials showed that *AREG* mRNA expression and AREG IHC were neither prognostic markers nor predictive markers of benefit from EGFR-targeted therapy in *RAS* wt metastatic colorectal cancer (Table 5; Fig. 7a, b). When examined as a continuous variable, neither *AREG* expression nor AREG IHC had prognostic significance for PFS and OS. In the predictive analysis of patients with *RAS* wt tumors, *AREG* expression predicted the effect of panitumumab treatment on PFS (HR 0.82, interaction *P* = 0.008) but not OS (HR 0.89, interaction test *P* = 0.07); and AREG IHC was not predictive of either PFS (HR 0.91, interaction test *P* = 0.06) or OS (HR 0.96, interaction test *P* = 0.43) (Table 5).

### Prognostic and Predictive role of other members of the HER family

Data on HER2 and HER4 were only available from the CALGB 80203 trial (Table 6). Prognostic analysis showed no significant effect of *HER2 and HER4* expression on PFS and OS in both the anti-EGFR therapy and control groups. The interaction test between treatment and gene expression also did not show a predictive value.

**Table 6:** Effect of HER for *RAS* wild-type patients, according to treatment

Biomarker	Study	Subgroup	N	PFS				OS			
				**Median, months**	**HR (95% CI)**	***P* value**	**Interaction *P***	**Median, months**	**HR (95% CI)**	***P* value**	**Interaction *P***
***HER2* mRNA**											
	**CALGB 80203**	**Cetuximab + FOLFOX or FOLFIRI (*N* = 26)**			0.67 (0.42, 1.08)	NR	0.77		0.86 (0.55, 1.33)	NR	0.62
		High (≥ the median)	NR	NR				NR			
		Low (< the median)	NR	NR				NR			
		**FOLFOX or FOLFIRI (*N* = 29)**			0.66 (0.39, 1.10)	NR			0.65 (0.39, 1.10)	NR	
		High (≥ the median)	NR	NR				NR			
		Low (< the median)	NR	NR				NR			
***HER4* mRNA**											
	**CALGB 80203**	**Cetuximab + FOLFOX or FOLFIRI (*N* = 26)**			0.63 (0.33, 1.16)	NR	0.076		0.77 (0.42, 1.37)	NR	0.07
		High (≥ the median)	NR	NR				NR			
		Low (< the median)	NR	NR				NR			
		**FOLFOX or FOLFIRI (*N* = 29)**			1.27 (0.79, 2.04)	NR			1.52 (0.95, 2.39)	NR	
		High (≥ the median)	NR	NR				NR			
		Low (< the median)	NR	NR				NR			
***HER3* mRNA**											
	**CALGB 80203**	**Cetuximab + FOLFOX or FOLFIRI (N = 26)**			0.98 (0.71, 1.36)	NR	0.20		1.15 (0.81, 1.62)	NR	0.029
		High (≥ the median)	NR	NR				NR			
		Low (< the median)	NR	NR				NR			
		**FOLFOX or FOLFIRI (*N* = 29)**			0.69 (0.43, 1.12)	NR			0.48 (0.27, 0.87)	NR	
		High (≥ the median)	NR	NR				NR			
		Low (< the median)	NR	NR				NR			
	**PICCOLO**	**Irinotecan (*N* = 115)**			0.98 (0.63, 1.52)	0.92			0.93 (0.60, 1.44)	0.74	
		High (> 66th centile)	33	NR				NR			
		Low (< 66th centile)	82	NR				NR			
		**Panitumumab + Irinotecan (*N* = 94)**			0.71 (0.61, 0.82)	< 0.001	0.003		0.73 (0.64, 0.83)	< 0.001	0.01
		Log2 HER3	94	NR							
		**Irinotecan (*N* = 115)**			0.91 (0.77, 1.07)	0.25			0.90 (0.80, 1.01)	0.07	
		Log2 HER3	115	NR				NR			

**Fig. 8:**
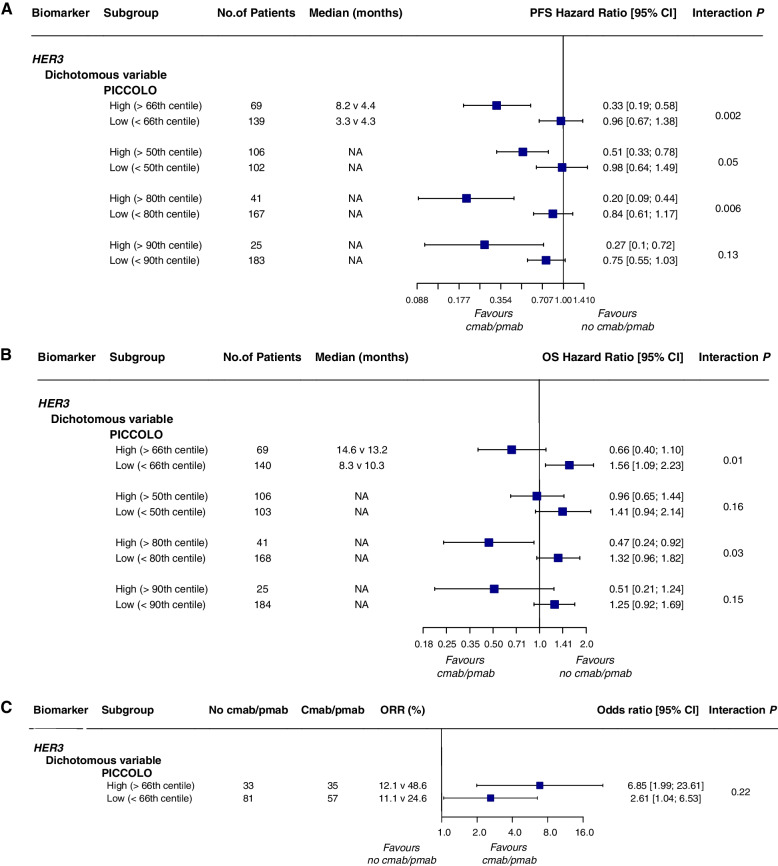
Forest plots for predictive analyses of HER3 in trials comparing experimental arm with control arm—**a** progression-free survival, **b** overall survival and **c** objective response rate. NA, not available; PFS, progression-free survival; OS overall survival; ORR, objective response rate

For HER3, neither of the two included trials (the PICCOLO and CALGB 80203 trials) observed evidence for it as a prognostic biomarker but found tumor *HER3* mRNA expression may be a useful predictive biomarker for anti-EGFR therapy in *RAS* wt patients (Table 6; Fig. 8a-c). However, the prediction of *HER3* expression levels in these two trials was in opposite directions. Considering the substantial statistical heterogeneity (*I*^2^ = 88%) associated with methodological and clinical characteristics, we ultimately decided not to perform a quantitative synthesis of the trials because it would be clinically meaningless, and the results would be difficult to interpret. In the CALGB 80203 trial, high *HER3* expression predicted a lack of OS benefit from cetuximab therapy [chemotherapy plus cetuximab: HR 1.15 (0.81–1.62); chemotherapy alone: HR 0.48 (0.27–0.87); interaction test *P* = 0.029] (Table 6). Conversely, in the PICCOLO trial, patients with high *HER3* mRNA expression significantly benefited from panitumumab, both as a continuous variable and a binary model. There was a significant interaction between biomarkers and treatment (continuous variable: HR 0.78, interaction test *P* = 0.003 for PFS; HR 0.81, interaction test *P* = 0.01 for OS, Table 6; dichotomous variable: HR 0.34, interaction test *P* = 0.002 for PFS; HR 0.42, interaction test *P* = 0.01 for OS, Fig. 8a, b).

### Prognostic and Predictive role of MicroRNA

**Fig. 9:**
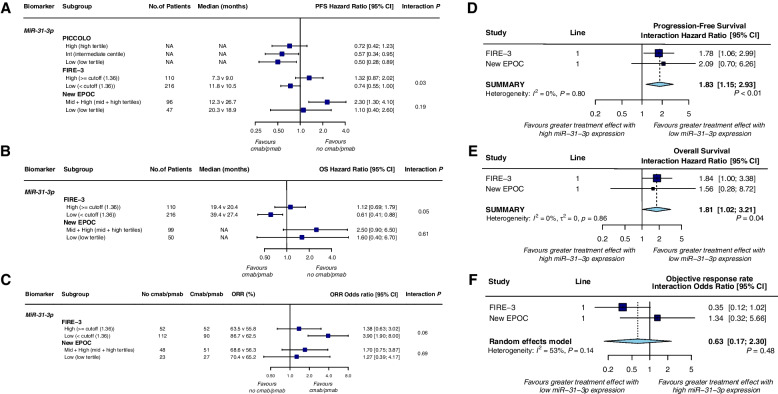
Forest plots for predictive analyses of miR-31-3p in trials comparing experimental arm with control arm—**a**, **d** progression-free survival, **b**, **e** overall survival and **c**, **f** objective response rate. NA, not available; PFS, progression-free survival; OS overall survival; ORR, objective response rate

Three trials assessed the value of relevant microRNAs as potential biomarkers. In the PICCOLO trial, a predefined model classified *RAS* wt patients (*n* = 188) into three tertiles, high, intermediate (int), and low *miR-31-3p* expression. Int and high expression patients had worse PFS (HR 1.60, 1.60; *P* = 0.018, respectively) and OS (HR 1.58, 2.03; *P* = 0.0012, respectively) compared with low expression patients and after adjustment for the treatment arm (Table 7), indicating a positive prognostic effect of low *miR-31-3p* expression. In predictive analysis, panitumumab produced a significant PFS benefit in patients with low and int *miR-31-3p* expression (HR 0.50; *P* = 0.019 and 0.57;* P* = 0.031, respectively) but not in patients with high expression (HR 0.72;* P* = 0.23) (Fig. 9a); however, no statistically significant treatment-expression interaction.

**Table 7:** Effect of MicroRNA for *RAS* wild-type patients, according to treatment

Biomarker	Study	Subgroup	N	PFS			OS			ORR		
				**Median, months**	**HR (95% CI)**	***P*** ** value**	**Median, months**	**HR (95% CI)**	***P*** ** value**	**Rate, %**	**Odds ratio (95% CI)**	***P*** ** value**
***MiR-31-3p***												
	**PICCOLO**											
		**Panitumumab ± Irinotecan (** ***N*** ** = 188)**				0.018			0.0012			NR
		Int: Low	NR	NR	1.60		NR	1.58		NR	NR	
		High: Low	NR	NR	1.60		NR	2.03		NR	NR	
	**FIRE-3**											
		**Cetuximab/Bevacizumab ± FOLFIRI (** ***N*** ** = 340)**			1.11 (0.85, 1.45)	0.43		1.43 (1.05, 1.96)	0.02		NR	NR
		High (≥ a prespecified cutoff (1.36))	111	7.8			20.3			NR		
		Low (< a prespecified cutoff (1.36))	229	11.1			30.3			NR		
		**Cetuximab/Bevacizumab ± FOLFIRI (** ***N*** ** = 340)**										
		Log miR-31-3p	340	NR	1.15 (1.05, 1.25)	< 0.01	NR	1.26 (1.13, 1.40)	< 0.01	NR	NR	NR
	**New EPOC**											
		**Cetuximab + oxaliplatin/irinotecan plus fluorouracil (** ***N*** ** = 78)**			2.00 (1.00, 4.20)	0.50		1.10 (0.40, 3.00)	0.85		0.92 (0.33, 2.54)	0.874
		Mid + High (mid + high tertiles)	51	12.3			NR			68.6		
		Low (low tertile)	27	20.3			NR			70.4		
		**Oxaliplatin/irinotecan plus fluorouracil (** ***N*** ** = 71)**			0.90 (0.40, 2.00)	0.79		0.70 (0.20, 2.90)	0.62		0.69 (0.25, 1.92)	0.473
		Mid + High (mid + high tertiles)	48	NR			NR			56.3		
		Low (low tertile)	23	NR			NR			65.2		
		**Cetuximab + oxaliplatin/irinotecan plus fluorouracil (** ***N*** ** = 62)**			1.20 (0.98, 1.48)	0.035		NR	NR		NR	NR
		Log2 miR-31-3p	62	NR			NR			NR		
		**Oxaliplatin/irinotecan plus fluorouracil (** ***N*** ** = 63)**			0.96 (0.75, 1.23)	0.36		NR	NR		NR	NR
		Log2 miR-31-3p	63	NR			NR			NR		

Data from the FIRE-3 trial not only showed the prognostic value of *miR-31-3p* expression but also observed a significant benefit of anti-EGFR mAb therapy in patients with low *miR-31-3p* expression tumors compared with no benefit in patients with high *miR-31-3p* expression tumors, but none of these results from the New EPOC trial were statistically significant (Table 7; Fig. 9a-c). Pooled results from two trials confirmed that *miR-31-3p* expression levels predicted PFS (HR = 1.83 (1.15, 2.93), interaction test *P* < 0.01; heterogeneity test *P* = 0.80, *I*^2^ = 0%, Fig. 9d) and OS (HR = 1.81 (1.02, 3.21), interaction test *P* = 0.04; heterogeneity test *P* = 0.86, *I*^2^ = 0%, Fig. 9e) benefits of anti-EGFR mAb therapy, but not for ORR (OR = 0.63 (0.17, 2.30), interaction test *P* = 0.48; heterogeneity test *P* = 0.14, *I*^2^ = 53%, Fig. 9f).

In addition, a study of patients from the FIRE-3 trial also suggested that miR-21 expression levels may be a predictive biomarker for anti-EGFR therapy (data not shown).

## Discussion

Colorectal cancer accounts for about one-tenth of global cancer and death cases, ranking third in incidence but second to lung cancer in terms of mortality [65]. Metastatic colorectal cancer is associated with a significantly worse prognosis, with a 5-year relative survival rate of no more than 15% for patients [66]. Since the US FDA approved cetuximab for the treatment of mCRC in 2004, EGFR-targeted therapy has become an essential means to improve the survival prognosis of *RAS* wt mCRC patients. However, primary and secondary resistance prevent many patients from benefiting from this therapy. Therefore, identifying other potential molecular biomarkers to guide this treatment and prognostic stratification of mCRC patients is highly desirable. We identified 30 publications in this systematic review involving 18 well-known RCTs, providing a comprehensive exploration of potential biomarkers currently widely studied.

The first to be extensively evaluated were critical components of the EGFR signaling pathway, including RAS, BRAF, PIK3CA, PTEN, and combinations of them [67]. This is driven by plausible biological rationale that constitutive activation of signaling pathways parallel to or downstream of EGFR should circumvent EGFR inhibition and therefore preclude sensitivity to anti-EGFR mAbs [68]. Some previous meta-analyses have highlighted the prognostic value of *BRAF* mutations, but there is insufficient evidence to demonstrate its predictive role for anti-EGFR mAbs therapy [26, 69, 70]. The efficacy of anti-EGFR mAb in patients with *BRAF*-mutated mCRC is still under debate. Recently, preliminary results from the randomized phase II FIRE-4.5 study (AIO KRK-0116) provided good data [71]. This is the first trial to investigate mFOLFOXIRI in combination with cetuximab or bevacizumab as first-line treatment for patients with *RAS* wild-type, *BRAF* V600E mutant mCRC. According to the results of the FIRE-4.5 trial presented at the 2021 ASCO Annual meeting, FOLFOXIRI plus cetuximab (49.2%) did not induce a higher ORR compared with FOLFOXIRI plus bevacizumab (60.0%); and both PFS and OS were significantly better in the bevacizumab group than in the cetuximab-treated group. This coincides with our results. Our pooled analysis of relevant data from 14 trials determined that *BRAF* mutation was not only a negative prognostic biomarker in patients with *RAS* wt tumors but also predicted a lack of benefit from anti-EGFR mAb therapy (interaction test *P* < 0.01 for PFS). Stratified analysis showed that the predictive effect of *BRAF* status on PFS might depend on cetuximab, first-line treatment, and the absence of bevacizumab in the control group. Although some studies have initially suggested that *RAS* wt mCRC patients with *PIK3CA* mutations and *PTEN* mutations or deletions may have lower responses and poorer outcomes when receiving anti-EGFR mAbs [72–76]. But we found no prognostic or predictive role for them based on the data from several available trials. However, a pooled analysis of two trials evaluating the combined biomarkers of *KRAS*, *NRAS*, *BRAF*, and *PIK3CA* showed a significantly reduced benefit from anti-EGFR therapy in patients with any mutant tumor compared with patients with all wt tumors (interaction tests *P* < 0.05 for PFS, OS, and ORR). This suggests that combinatorial analysis of multiple biomarkers can help further screen outpatient populations that may benefit.

EGFR and its ligands EREG and AREG are commonly overexpressed in colorectal cancer [77]. Autocrine stimulation of EGFR by AREG and EREG is a mechanism of tumor EGFR pathway dependence, so the impact of their expression on the response to EFGR-targeted therapy in mCRC patients has also received much attention [78–80]. Several retrospective studies have suggested that EGFR status and EREG/AREG expression may correlate with the prognosis of mCRC patients and may identify patients who will or will not benefit from anti-EGFR therapy [16, 80–86]. However, the strength of the evidence is limited, and the conclusions are controversial, so further validation of the clinical utility of these markers is needed [23]. Under this topic, we only performed a descriptive analysis of relevant trial data because of the high heterogeneity among studies. EGFR was targeted in six trials involving its immunohistochemical protein expression, mRNA expression, and frequency of gene amplification. No prognostic association has been found between this parameter and survival outcomes in *RAS* wt patients. In terms of predictive effects, data from the 20,020,408 and PICCOLO trials suggest that patients with increased *EGFR* GCN had higher response rates to panitumumab and longer PFS; however, there was no evidence of a predictive role for EGFR protein and mRNA expression. Four trials analyzed EREG and AREG alone or as a combined biomarker. For EREG/AREG as a combined dichotomous biomarker, high ligand mRNA levels or IHC positivity could predict PFS and ORR benefit from panitumumab treatment, but no prognostic effect was observed. When EREG and AREG were investigated as separate biomarkers, conflicting results emerged, and EREG expression may have certain prognostic and predictive roles compared with AREG. Still, no clear conclusions can be drawn at this time.

The HER family of receptor tyrosine kinases shares a high degree of structural and functional homology, which is the molecular basis for receptor interaction and cross-activation [87]. Dimers containing HER3, especially the HER2-HER3 heterodimer, have been shown to act as oncogenic units to drive tumor cell proliferation [88]. And preclinical experiments showed that cetuximab induces HER2-HER3 dimers in colon cancer cells [89].Several small clinical reports support the importance of expression of other markers in the HER axis, suggesting that *HER2* amplification and *HER3* overexpression appear to be predictive markers of resistance to anti-EGFR mAb therapy [90–94]. Of the included trials, only two provided data on *HER2*, *HER3*, and *HER4* expression. In prognostic analysis, no meaningful results were observed for all three. In terms of predictive effect, only tumor *HER3* mRNA expression may be a predictive biomarker for anti-EGFR therapy in *RAS* wt patients. However, in contrast to the results of previous retrospective studies and the CALGB 80203 trial, the PICCOLO trial showed that *HER3* overexpression was significantly associated with benefit rather than resistance to anti-EGFR therapy. This result may be due to the role of HER3 as an obligate heterodimer. Overall, further studies are needed to verify the role of other members of the HER family.

MicroRNAs (miRNAs) are a group of short non-coding RNAs that play important roles in carcinogenesis and tumor progression, and their aberrant expression may potentially be used as diagnostic, therapeutic, and prognostic markers for CRC [95–98]. MiR-31 is frequently upregulated in CRC tumors compared with normal mucosa and miR-31 activates the RAS pathway and functions by repressing RAS p21 GTPase activating protein 1 translation, thereby promoting CRC progression [99, 100]. In addition, it has been shown that low expression of miR-31-3p could be a consequence of the regulation of pre-mir-31 maturation by an EGFR-activated pathway, driving tumor sensitivity to anti-EGFR therapy [58].Our meta-analysis showed that low *miR-31-3p* expression predicted PFS (interaction test *P* = 0.01) and OS benefit (interaction test *P* = 0.04) from anti-EGFR mAb treatment. In terms of prognostic analysis, low *miR-31-3p* expression also showed a positive prognostic effect. In addition, a trial assessing the expression level of *miR-21* also showed preliminary predictive value.

To our knowledge, this is the first systematic review to systematically summarize the evidence from RCTs on the prognostic and predictive value of all extensively studied potential biomarkers of interest, and the largest on this topic. However, the current analysis also has some limitations that must be acknowledged. First, the results of the trials we included in the analysis were extracted from published data and not based on individual patient data. Second, some biomarkers were only analyzed descriptively with limited strength of evidence due to limitations in the number of included trials and the high heterogeneity caused by differences in assay methods, determination criteria, etc. Furthermore, stratified analyzes of interest could not be performed in each marker due to the limited number of studies. Moreover, some factors that may influence the impact of mutations on survival, such as MSI status, were limited by the fact that the original report did not adjust the hazard ratio for them, and could not be further analyzed. Finally, some of the studies included in the analysis were only reported as abstracts in conference presentations rather than fully published articles, and results may differ slightly between future full publications.

## Conclusions

In conclusion, our analysis was able to confirm that, in *RAS* wt mCRC patients, *BRAF* mutations were associated with poor prognosis and predicted lack of response to anti-EGFR therapy, and combination biomarker *KRAS*/*NRAS*/*BRAF*/*PIK3CA* mutations were also negative predictive markers for treatment; low *miR-31-3p* expression was positive prognostic and predictive of therapy. There is currently insufficient evidence to support *PIK3CA* mutations, *PTEN* mutations or deletions, EGFR status, and *HER2* and *HER4* expression as prognostic or therapy predictive biomarkers. EREG/AREG and *HER3* expression may have a particular predictive role, but the conclusions are still controversial. These results are preliminary, and efforts are needed to achieve assay standardization and prospective validation to optimize further the identification of patients who will benefit from anti-EGFR therapy.

### Supplementary Information

**Additional file 1:** **Appendix A.** Detailed search strategy

** Additional file 2: Supplementary Table S1. **PRISMA checklist.

** Additional file 3:** **Supplementary Table S2.** 30 publications included in the review.

** Additional file 4:** **Supplementary Table S3.** Risk of Bias Assessment using the Newcastle-Ottawa Scale^a^.

## Data Availability

The datasets supporting the conclusions of this article are included within the article and its additional files.
