# Supplementary material for: Prognostic and predictive biomarkers for anti-EGFR monoclonal antibody therapy in RAS wild-type metastatic colorectal cancer: a systematic review and meta-analysis
Source: BMC Cancer. 2023 Nov 16;23:1117. doi: 10.1186/s12885-023-11600-z (PMC10655341; doi:10.1186/s12885-023-11600-z)
Supplement: Supplementary file 2 — Additional file 2: Supplementary Table S1. PRISMA checklist. [file 12885_2023_11600_MOESM2_ESM.docx]

**Supplementary Table S2.** 30 publications included in the review.

| **N.** | **Study** | **Type of Study** | **Anti-EGFR agent  vs Comparator** | **Line,  Background Therapy** | **ITT, N** | **With *RAS* Evaluable** | | **With *RAS* wt** | **Biomarker** | | | | |
| --- | --- | --- | --- | --- | --- | --- | --- | --- | --- | --- | --- | --- | --- |
|  |  |  |  |  |  |  |  |  | **Method** | **Evaluated, N of *RAS* wt** | | **Positive, N** | |
| **1** | Peeters 2013 20020408 | Prospective using archived samples | Panitumumab vs  No Panitumumab | ≥ 3rd line,  BSC | 463 | *KRAS* codons 12, 13, and 61 | 288 | 153 | Next-generation sequencing | *BRAF* exon 15 | 130 | Mutation | 15 |
|  |  |  |  |  |  |  |  |  | Next-generation sequencing | *PIK3CA* exons 1, 2, 9, 10, and 20 | 138 | Mutation | 10 |
|  |  |  |  |  |  | \|  \| \| --- \| |  |  | Next-generation sequencing | *PTEN* | 144 | Mutation | 9 |
| **2** | Sartore-Bianchi 2007 20020408 | Prospective using archived samples | Panitumumab vs  No Panitumumab | ≥ 3rd line,  BSC | 463 | NA | NA | NA | FISH | *EGFR*GCN | 92 | ≥ 2.5/nucleus | 27 |
|  |  |  |  |  |  |  |  |  | FISH | *EGFR* chromosome 7 polysomy | 58 | ≥ 40% | 22 |
| **3** | Peeters 2015 20050181 | Prospective using archived samples | Panitumumab vs  No Panitumumab | 2nd line,  FOLFIRI | 1186 | *KRAS* and *NRAS*  exons 2, 3, and 4 | 1014 | 421 | Sanger sequencing | *BRAF* exon 15 | 421 | Mutation | 45 |
| **4** | Kim 2018 20100007 | Prospective using archived samples | Panitumumab vs  No Panitumumab | 2nd line,  BSC | 377 | *KRAS* and *NRAS*  exons 2, 3, and 4 | 377 | 270 | Sanger sequencing | *BRAF* exon 15 | 270 | Mutation | 20 |
| **5** | Bokemeyer 2012 CRYSTAL and OPUS | Prospective using archived samples | Cetuximab vs  No Cetuximab | 1st line,  FOLFIRI (CRYSTAL) or FOLFOX-4 (OPUS) | 1535 | *KRAS* codons 12 and 13 of exon 2 | 1378 | 845 | PCR clamping and melting curve analysis | *BRAF* V600E | 800 | Mutation | 70 |
| **6** | Douillard 2013 PRIME | Prospective using archived samples | Panitumumab vs  No Panitumumab | 1st line,  FOLFOX4 | 1183 | *KRAS* and *NRAS*  exons 2, 3, and 4 | 1060 | 512 | Sanger sequencing | *BRAF* exon 15 | 499 | Mutation | 53 |
| **7** | Karapetis 2014 CO.17 | Prospective using archived samples | Cetuximab vs  No Cetuximab | ≥ 2nd line,  BSC | 572 | *KRAS* exon 2 | 394 | 230 | Nested PCR and sequencing | *BRAF* exon 15 | 208 | Mutation | 10 |
|  |  |  |  |  |  |  |  |  | Nested PCR and high-resolution melting analysis | *PIK3CA* exons 9 and 20 | 211 | Mutation | 26 |
|  |  |  |  |  |  |  |  |  | IHC | PTEN | 116 | Negative | 83 |
| **8** | Qin 2018 TAILOR | Prospective using archived samples | Cetuximab vs  No Cetuximab | 1st line,  FOLFOX4 | 393 | *KRAS* and *NRAS*  exons 2, 3, and 4 | 393 | 393 | NA | *BRAF* | 393 | Mutation | 52 |
|  |  |  |  |  |  |  |  |  | NA | EGFR-positive cytoplasmic intensity | 354 | 0 | 244 |
|  |  |  |  |  |  |  |  |  |  |  |  | 1+ | 46 |
|  |  |  |  |  |  |  |  |  |  |  |  | 2+ | 39 |
|  |  |  |  |  |  |  |  |  |  |  |  | 3+ | 25 |
| **9** | Seymour 2013 PICCOLO | Prospective using archived samples | Panitumumab vs  No Panitumumab | 2nd line,  Irinotecan | 696 | *KRAS* codons 12, 13, and 61 | 523 | 523 | Pyrosequencing | *BRAF* V600E | 460 | Mutation | 68 |
|  |  |  |  |  |  |  |  |  | Pyrosequencing | *PIK3CA* exons 9 and 20 | 460 | Mutation | 32 |
|  |  |  |  |  |  |  |  |  | Pyrosequencing | *KRAS*/*NRAS*/*BRAF*/*PIK3CA* | 460 | Any-mutation | 137 |
| **10** | Smith 2013 COIN | Prospective using archived samples | Cetuximab vs  No Cetuximab | 1st line,  Oxaliplatin and fluoropyrimidine | 2245 | *KRAS* codons 12, 13, and 61 | 1,949 | 1,125 | Pyrosequencing and Sequenom | *BRAF* (D594G and V600E) | 1061 | Mutation | 170 |
|  |  |  |  |  |  |  |  |  | Pyrosequencing and Sequenom | *PIK3CA* exon 9 | 1101 | Mutation | 74 |
|  |  |  |  |  |  |  |  |  | Pyrosequencing and Sequenom | *PIK3CA* exon 20 | 1101 | Mutation | 40 |
| **11** | Maughan 2011 COIN | Prospective using archived samples | Cetuximab vs  No Cetuximab | 1st line,  Oxaliplatin and fluoropyrimidine | 1630 | *KRAS* codons 12, 13, 61 and *NRAS* codons 12, 61 | 1316 | 690 | Pyrosequencing and Sequenom | *BRAF* (D594G and V600E) | 690 | Mutation | 102 |
|  |  |  |  |  |  |  |  |  | IHC | EGFR membrane staining cells | NA | ≥ 10% | 639* |
| **12** | Guren 2017 NORDIC-VII | Prospective using archived samples | Cetuximab vs  No Cetuximab | 1st line,  FLOX | 566 | *KRAS* and *NRAS*  exons 2, 3, and 4 | 457 | 247 | ARMS-PCR | *BRAF* V600E | 247 | Mutation | 55 |
| **13** | Laurent-Puig 2019 FIRE-3 | Prospective using archived samples | Cetuximab vs  Bevacizumab | 1st line,  FOLFIRI | 592 | *KRAS* and *NRAS*  exons 2, 3, and 4 | 515 | 343 | NA | *BRAF* V600E | 340 | Mutation | 42 |
|  |  |  |  |  |  |  |  |  | qPCR | *MiR-31-3p* | 340 | High  (≥ 1.36) | 111 |
| **14** | Innocenti 2019 CALGB/SWOG 80405 | Prospective using archived samples | Cetuximab vs  Bevacizumab | 1st line,  mFOLFOX6 or FOLFIRI | 2326 | *KRAS* codons 12 and 13 | 843 | 600 | Allele-specific PCR / AS-PCR | *BRAF* | 504 | Mutation | 72 |
| **15** | Rivera 2017 PEAK | Prospective using archived samples | Panitumumab vs  Bevacizumab | 1st line,  mFOLFOX6 | 285 | *KRAS* and *NRAS*  exons 2, 3, and 4 | 250 | 170 | Sanger sequencing | *BRAF* V600E | 170 | Mutation | 14 |
| **16** | Ciardiello 2016 CAPRI-GOIM | Prospective using archived samples | Cetuximab vs  No Cetuximab | 2nd line,  FOLFOX | 153 | *KRAS* exon 2 | 153 | 153 | Next-generation sequencing | *KRAS*/*NRAS*/*BRAF*/*PIK3CA* | 117 | Any-mutation | 51 |
| **17** | Sastre 2021 VISNÚ-2 | Prospective | Cetuximab vs  Bevacizumab | 1st line,  FOLFIRI | 240 | *KRAS* exons 2 and 3 | 240 | 240 | Cobas® test | *BRAF*/*PIK3CA* | 240 | Any-mutation | 44 |
| **18** | Seligmann 2017 PICCOLO | Prospective using archived samples | Panitumumab vs  No Panitumumab | 2nd line,  Irinotecan | 460 | *RAS* | 275 | 234 | Affymetrix OncoScan array | *EGFR* CN | NA | Gain (> 2 copies) | 196* |
| **19** | Licitra 2013 CRYSTAL | Prospective using archived samples | Cetuximab vs  No Cetuximab | 1st line,  FOLFIRI | 1198 | *KRAS* | 1058 | 664 | IHC | EGFR IHC score | 664 | 0–300 scale | 664 |
| **20** | Qin 2016 TAILOR | Prospective using archived samples | Cetuximab vs  No Cetuximab | 1st line,  FOLFOX4 | 393 | *KRAS* and *NRAS*  exons 2, 3, and 4 | 393 | 393 | IHC | EGFR-positive cells % | 354 | 0% | 162 |
|  |  |  |  |  |  |  |  |  |  |  |  | > 0%-10% | 83 |
|  |  |  |  |  |  |  |  |  |  |  |  | > 10%-20% | 29 |
|  |  |  |  |  |  |  |  |  |  |  |  | > 20%-35% | 23 |
|  |  |  |  |  |  |  |  |  |  |  |  | > 35% | 57 |
| **21** | Cushman 2015 CALGB 80203 | Prospective using archived samples | Cetuximab vs No Cetuximab | 1st line,  FOLFOX or FOLFIRI | 238 | *KRAS*codons 12 and 13 | 103 | 55 | qPCR | *EGFR*mRNA | 55 | High (≥ the median) | NA |
|  |  |  |  |  |  |  |  |  | qPCR | *EREG* mRNA | 55 | High (≥ the median) | NA |
|  |  |  |  |  |  |  |  |  | qPCR | *AREG* mRNA | 55 | High (≥ the median) | NA |
|  |  |  |  |  |  |  |  |  | qPCR | *HER2* mRNA | 55 | High (≥ the median) | NA |
|  |  |  |  |  |  |  |  |  | qPCR | *HER3* mRNA | 55 | High (≥ the median) | NA |
|  |  |  |  |  |  |  |  |  | qPCR | *HER4* mRNA | 55 | High (≥ the median) | NA |
| **22** | Jonker 2014 CO.17 | Prospective using archived samples | Cetuximab vs  No Cetuximab | ≥ 2nd line,  BSC | 572 | *KRAS* | 394 | 230 | qPCR | *EREG* mRNA | 225 | High (prespecified threshold) | 139 |
|  |  |  |  |  |  |  |  |  |  |  |  | High (minimum *P*-value threshold) | 169 |
|  |  |  |  |  |  |  |  |  | qPCR | *EREG* mRNA | 225 | Continuous variable | 225 |
| **23** | Adams 2012 COIN | Prospective using archived samples | Cetuximab vs  No Cetuximab | 1st line,  Oxaliplatin and fluoropyrimidine | 1630 | NA | 952 | NR | RT-PCR | *EREG* mRNA | NA | High (80% centile) | NA |
|  |  |  |  |  |  |  |  |  |  |  |  | High (50% centile) | NA |
| **24** | Williams 2021 PICCOLO | Prospective using archived samples | Panitumumab vs  No Panitumumab | 2nd line,  Irinotecan | 460 | *KRAS* c.12,13,59-61,146 and *NRAS* c.12,13,59-61 | 313 | 274 | IHC | EREG | 274 | High (>50%) | 117 |
|  |  |  |  |  |  |  |  |  | IHC | EREG | 274 | Continuous variable | 274 |
|  |  |  |  |  |  |  |  |  | IHC | AREG | 274 | High (>50%) | 86 |
|  |  |  |  |  |  |  |  |  | IHC | AREG | 274 | Continuous variable | 274 |
|  |  |  |  |  |  |  |  |  | IHC | AREG/EREG | 274 | High (> 50% AREG or > 50% EREG) | 132 |
| **25** | Seligmann 2016 PICCOLO | Prospective using archived samples | Panitumumab vs  No Panitumumab | 2nd line,  Irinotecan | 696 | *KRAS* c.12,13,61,  146 and *NRAS* c.12,13,61 | 323 | 220 | RT-PCR | *EREG* mRNA | 220 | Log EREG | 220 |
|  |  |  |  |  |  |  |  |  | RT-PCR | *AREG* mRNA | 220 | Log EREG | 220 |
|  |  |  |  |  |  |  |  |  | RT-PCR | *EREG/AREG* mRNA | 220 | High (either EREG or AREG in top tertile) | 99 |
| **26** | Seligmann 2018 PICCOLO | Prospective using archived samples | Panitumumab vs  No Panitumumab | 2nd line,  Irinotecan | 696 | *KRAS* c.12,13,61,  146 and *NRAS* c.12,13,61 | 308 | 209 | RT-PCR | *HER3* mRNA | 209 | High (> 66th centile) | 69 |
|  |  |  |  |  |  |  |  |  | RT-PCR | *HER3* mRNA | 209 | Log2 HER3 | 209 |
| **27** | Laurent-Puig 2015 PICCOLO | Prospective using archived samples | Panitumumab vs  No Panitumumab | 2nd line,  Irinotecan | 696 | *KRAS* and *NRAS* | 213 | 188 | NA | *MiR-31-3p* | 188 | High tertile | NA |
|  |  |  |  |  |  |  |  |  |  |  |  | Intermediate (int) | NA |
|  |  |  |  |  |  |  |  |  |  |  |  | Low tertile | NA |
| **28** | Pugh 2017 New EPOC | Prospective using archived samples | Cetuximab vs  No Cetuximab | 1st line,  oxaliplatin/irinotecan plus fluorouracil | 257 | *KRAS* codons 12, 13, and 61 | 257 | 257 | qPCR | *MiR-31-3p* | 149 | High tertile | 50 |
|  |  |  |  |  |  |  |  |  |  |  |  | Intermediate tertile | 49 |
|  |  |  |  |  |  |  |  |  |  |  |  | Low tertile | 50 |
| **29** | Laurent-Puig 2014 New EPOC | Prospective using archived samples | Cetuximab vs  No Cetuximab | 1st line,  oxaliplatin/irinotecan plus fluorouracil | 257 | *KRAS* codons 12, 13, and 61 | 257 | 257 | qPCR | *MiR-31-3p* | 125 | Log2 miR-31-3p | 125 |
| **30** | Miller-Phillips 2019 FIRE-3 | Prospective using archived samples | Cetuximab vs  Bevacizumab | 1st line,  FOLFIRI | 592 | *KRAS* exon 2 | 333 | 333 | RT-PCR | *MiR-21* | 333 | High (≥ the median) | NA |
