# Supplementary material for: Prognostic and predictive biomarkers for anti-EGFR monoclonal antibody therapy in RAS wild-type metastatic colorectal cancer: a systematic review and meta-analysis
Source: BMC Cancer. 2023 Nov 16;23:1117. doi: 10.1186/s12885-023-11600-z (PMC10655341; doi:10.1186/s12885-023-11600-z)
Supplement: Supplementary file 3 — Additional file 3: Supplementary Table S2. 30 publications included in the review. [file 12885_2023_11600_MOESM3_ESM.docx]

**Supplementary Table S3.** Risk of Bias Assessment using the Newcastle-Ottawa Scale^a^.

|  | **Selection** | | | |  |  | **Comparability** |  | **Outcome** | | |  |  |
| --- | --- | --- | --- | --- | --- | --- | --- | --- | --- | --- | --- | --- | --- |
| **Study** | **Representativeness of the exposed cohort** | **Selection of the non-exposed cohort** | **Ascertainment of exposure** | **Demonstration that outcome of interest was not present at start of study** |  |  | **Comparability of cohorts on the basis of the design or analysis** |  | **Assessment of outcome** | **Was follow up long enough for outcomes to occur** | **Adequacy of follow up of cohorts** | **Scores** | **Overall study quality** |
| Peeters 2013  20020408 | * | * | * | * |  |  | ** |  | * | * | * | 9 | Good |
| Sartore-Bianchi 2007  20020408 | * | * | * | * |  |  | - |  | * | * | * | 7 | Fair |
| Peeters 2015  20050181 | * | * | * | * |  |  | ** |  | * | * | * | 9 | Good |
| Kim 2018  20100007 | * | * | * | * |  |  | ** |  | * | * | * | 9 | Good |
| Bokemeyer 2012  CRYSTAL and OPUS | * | * | * | * |  |  | * |  | * | * | * | 8 | Good |
| Douillard 2013  PRIME | * | * | * | * |  |  | ** |  | * | * | * | 9 | Good |
| Karapetis 2014  CO.17 | * | * | * | * |  |  | ** |  | * | * | * | 9 | Good |
| Qin 2018  TAILOR | * | * | - | * |  |  | - |  | * | * | * | 6 | Fair |
| Seymour 2013  PICCOLO | * | * | * | * |  |  | ** |  | * | * | * | 9 | Good |
| Smith 2013  COIN | * | * | * | * |  |  | - |  | * | * | * | 7 | Fair |
| Maughan 2011  COIN | * | * | * | * |  |  | - |  | * | * | * | 7 | Fair |
| Guren 2017  NORDIC-VII | * | * | * | * |  |  | - |  | * | * | * | 7 | Fair |
| Laurent-Puig 2019  FIRE-3 | * | * | * | * |  |  | ** |  | * | * | * | 9 | Good |
| Innocenti 2019  CALGB/SWOG 80405 | * | * | * | * |  |  | ** |  | * | * | * | 9 | Good |
| Rivera 2017  PEAK | * | * | * | * |  |  | * |  | * | * | * | 8 | Good |
| Ciardiello 2016  CAPRI-GOIM | * | * | * | * |  |  | - |  | * | * | * | 7 | Fair |
| Sastre 2021 ^b^ VISNÚ-2 | * | * | * | - |  |  | - |  | * | * | * | 6 | Fair |
| Seligmann 2017  PICCOLO | * | * | * | * |  |  | - |  | * | * | * | 7 | Fair |
| Licitra 2013  CRYSTAL | * | * | * | * |  |  | - |  | * | * | * | 7 | Fair |
| Qin 2016  TAILOR | * | * | * | * |  |  | - |  | * | * | * | 7 | Fair |
| Cushman 2015  CALGB 80203 | * | * | * | * |  |  | - |  | * | * | * | 7 | Fair |
| Jonker 2014  CO.17 | * | * | * | * |  |  | * |  | * | * | * | 8 | Good |
| Adams 2012  COIN | * | * | * | * |  |  | - |  | * | * | * | 7 | Fair |
| Williams 2021  PICCOLO | * | * | * | * |  |  | * |  | * | * | * | 8 | Good |
| Seligmann 2016 PICCOLO | * | * | * | * |  |  | ** |  | * | * | * | 9 | Good |
| Seligmann 2018  PICCOLO | * | * | * | * |  |  | * |  | * | * | * | 8 | Good |
| Laurent-Puig 2015  PICCOLO | * | * | - | * |  |  | * |  | * | * | * | 7 | Fair |
| Pugh 2017  New EPOC | * | * | * | * |  |  | - |  | * | * | * | 7 | Fair |
| Laurent-Puig 2014  New EPOC | * | * | * | * |  |  | * |  | * | * | * | 8 | Good |
| Miller-Phillips 2019  FIRE-3 | * | * | * | * |  |  | - |  | * | * | * | 7 | Fair |

a Good quality: > 7 stars; Fair quality: 5-7 stars; Poor quality: < 5 stars; b Prospective randomized controlled trial designed to address tumor marker.
