# Supplementary material for: Prognostic and predictive biomarkers for anti-EGFR monoclonal antibody therapy in RAS wild-type metastatic colorectal cancer: a systematic review and meta-analysis
Source: BMC Cancer. 2023 Nov 16;23:1117. doi: 10.1186/s12885-023-11600-z (PMC10655341; doi:10.1186/s12885-023-11600-z)
Supplement: Supplementary file 4 — Additional file 4: Supplementary Table S3. Risk of Bias Assessment using the Newcastle-Ottawa Scalea. [file 12885_2023_11600_MOESM4_ESM.docx]

**Appendix A. Detailed search strategy**

**1. Pubmed**

| **Search** | **Most Recent Queries** | **Result** |
| --- | --- | --- |
| #18 | Search: #16 NOT #17 | 1830 |
| #17 | Search: #15 Limits: Humans, Editorial, Letter, Review | 926 |
| #16 | Search: #15 Limits: Humans | 2,756 |
| #15 | Search: #5 AND #9 AND #14 | 3,257 |
| #14 | Search: #10 OR #11 OR #12 OR #13 | 350,126 |
| #13 | Search: (colorectal neoplasm) or (colorectal tumor) or (colorectal cancer) or (colorectal carcinoma) or (colonic neoplasm) or (colonic cancer) or (colon neoplasm) or (colon cancer) or (colon adenocarcinoma) or (colon carcinoma) or (rectal neoplasm) or (rectal tumor) or (rectal cancer) or (rectal carcinoma) or (rectum neoplasm) or (rectum cancer) or (rectum carcinoma) or (CRC) | 350,126 |
| #12 | Search: Rectal Neoplasms[MeSH Terms] | 50,858 |
| #11 | Search: Colonic Neoplasms[MeSH Terms] | 78,983 |
| #10 | Search: Colorectal Neoplasms[MeSH Terms] | 220,858 |
| #9 | Search: #6 OR #7 OR #8 | 129,214 |
| #8 | Search: (epidermal growth factor receptor) or (EGF receptor) or (EGFR) or (ErbB-1) or (HER1) | 129,214 |
| #7 | Search: Genes, erbB-1[MeSH Terms] | 1,312 |
| #6 | Search: ErbB Receptors[MeSH Terms] | 68,595 |
| #5 | Search: #1 OR #2 OR #3 OR #4 | 367,874 |
| #4 | Search: (monoclonal antibody) OR (monoclonal antibodies) OR (mAb) OR (MoAb) or (Cetuximab) or (erbitux) or (c225) or (c-225) or (Panitumumab) or (ABX-EGF) or (vectibix) | 367,874 |
| #3 | Search: Panitumumab[MeSH Terms] | 1,094 |
| #2 | Search: Cetuximab[MeSH Terms] | 4,980 |
| #1 | Search: Antibodies, Monoclonal[MeSH Terms] | 259,656 |

**2. Embase**

| **#** | **Searches** | **Results** |
| --- | --- | --- |
| #17 | #16 AND [humans]/lim | 5,179 |
| #16 | #14 NOT #15 | 5,703 |
| #15 | #14 AND ([editorial]/lim OR [letter]/lim OR [review]/lim) | 2,346 |
| #14 | #5 AND #8 AND #13 | 8,049 |
| #13 | #9 OR #10 OR #11 OR #12 | 408,960 |
| #12 | 'colorectal neoplasm':ti,ab,kw OR 'colorectal tumor':ti,ab,kw OR 'colorectal cancer':ti,ab,kw OR 'colorectal carcinoma':ti,ab,kw OR 'colonic neoplasm':ti,ab,kw OR 'colonic cancer':ti,ab,kw OR 'colon neoplasm':ti,ab,kw OR 'colon cancer':ti,ab,kw OR 'colon adenocarcinoma':ti,ab,kw OR 'colon carcinoma':ti,ab,kw OR 'rectal neoplasm':ti,ab,kw OR 'rectal tumor':ti,ab,kw OR 'rectal cancer':ti,ab,kw OR 'rectal carcinoma':ti,ab,kw OR 'rectum neoplasm':ti,ab,kw OR 'rectum cancer':ti,ab,kw OR 'rectum carcinoma':ti,ab,kw OR crc:ti,ab,kw | 304,386 |
| #11 | 'rectum cancer'/exp | 250,218 |
| #10 | 'colon cancer'/exp | 304,162 |
| #9 | 'colorectal cancer'/exp | 204,959 |
| #8 | #6 OR #7 | 187,423 |
| #7 | 'epidermal growth factor receptor':ti,ab,kw OR 'egf receptor':ti,ab,kw OR egfr:ti,ab,kw OR 'erbb-1':ti,ab,kw OR her1:ti,ab,kw | 164,576 |
| #6 | 'epidermal growth factor receptor'/exp | 93,212 |
| #5 | #1 OR #2 OR #3 OR #4 | 751,113 |
| #4 | 'monoclonal antibody':ti,ab,kw OR 'monoclonal antibodies':ti,ab,kw OR mab:ti,ab,kw OR moab:ti,ab,kw OR cetuximab:ti,ab,kw OR erbitux:ti,ab,kw OR c225:ti,ab,kw OR 'c-225':ti,ab,kw OR 'abx-egf':ti,ab,kw OR panitumumab:ti,ab,kw | 282,791 |
| #3 | 'panitumumab'/exp | 8,887 |
| #2 | 'cetuximab'/exp | 30,803 |
| #1 | 'monoclonal antibody'/exp | 668,595 |

**3. The Cochrane Library**

| **ID** | **Search** | **Hits** |
| --- | --- | --- |
| #1 | MeSH descriptor: [Antibodies, Monoclonal] explode all trees | 14985 |
| #2 | MeSH descriptor: [Cetuximab] explode all trees | 649 |
| #3 | MeSH descriptor: [Panitumumab] explode all trees | 105 |
| #4 | ("monoclonal antibody") OR ("monoclonal antibodies") OR (mAb) OR (MoAb) or (Cetuximab) or (erbitux) or (c225) or ("c-225") or (Panitumumab) or ("ABX-EGF") or (vectibix) | 16899 |
| #5 | #1 OR #2 OR #3 OR #4 | 26796 |
| #6 | MeSH descriptor: [ErbB Receptors] explode all trees | 1459 |
| #7 | MeSH descriptor: [Genes, erbB-1] explode all trees | 31 |
| #8 | ("epidermal growth factor receptor") or ("EGF receptor") or (EGFR) or ("ErbB-1") or (HER1) | 13470 |
| #9 | #6 OR #7 OR #8 | 13954 |
| #10 | MeSH descriptor: [Colorectal Neoplasms] explode all trees | 8938 |
| #11 | MeSH descriptor: [Colonic Neoplasms] explode all trees | 1855 |
| #12 | MeSH descriptor: [Rectal Neoplasms] explode all trees | 1956 |
| #13 | ("colorectal neoplasm") or ("colorectal tumor") or ("colorectal cancer") or ("colorectal carcinoma") or ("colonic neoplasm") or ("colonic cancer") or ("colon neoplasm") or ("colon cancer") or ("colon adenocarcinoma") or ("colon carcinoma") or ("rectal neoplasm") or ("rectal tumor") or ("rectal cancer") or ("rectal carcinoma") or ("rectum neoplasm") or ("rectum cancer") or ("rectum carcinoma") or (CRC) | 21578 |
| #14 | #10 OR #11 OR #12 OR #13 | 23397 |
| #15 | #5 AND #9 AND #14 | 629 |
